# Community Risk, Mobility Patterns, and COVID-19 Hospitalization Risk in Texas: ZIP Code–Level Ecological Longitudinal Study

**DOI:** 10.2196/97405

**Published:** 2026-11-04

**Authors:** Jose Herrera-Diestra, Dongah Kim, Shraddha Ramdas Bandekar, Emily Javan, Spencer J Fox, Remy Pasco, Lauren Ancel Meyers

**Affiliations:** 1 Department of Mathematics, Tarleton State University, 1333 W Washington St, Stephenville, TX, 76401, United States, 1 (254) 968-9000; 2 Department of Integrative Biology, The University of Texas at Austin, Austin, TX, United States; 3 School of Informatics, Computing, and Cyber Systems, Northern Arizona University, Flagstaff, AZ, United States; 4 BioFrontiers Institute, University of Colorado Boulder, Boulder, CO, United States

**Keywords:** digital epidemiology, mobility data, COVID-19 hospitalization, community risk, behavioral response, spatial heterogeneity

## Abstract

**Background:**

The COVID-19 pandemic revealed substantial disparities in health outcomes across US communities. High-resolution mobility data offer new opportunities for real-time surveillance of behavioral responses, yet less is known about how precautionary behavior varied within cities and how these differences were associated with subsequent COVID-19 hospitalization risk.

**Objective:**

This study assessed how community-level risk shapes heterogeneity in precautionary mobility behavior within cities and whether these differences are associated with subsequent COVID-19 hospitalization risk.

**Methods:**

We analyzed anonymized mobile device data from 623 ZIP codes in the 20 most populous counties in Texas and COVID-19 hospitalization records comprising 65,474 hospital admissions from 620 ZIP codes (February-July 2020). We constructed a ZIP code–level measure of precautionary mobility reduction (PMR) and linked it to a composite community risk score derived from US Centers for Disease Control and Prevention social vulnerability indicators. Generalized additive mixed models estimated associations among community risk, mobility patterns, and weekly hospitalization rates, adjusting for epidemic timing, population size, and spatial clustering.

**Results:**

Mobility declined by approximately 73% relative to baseline following statewide stay-at-home orders but varied substantially across ZIP codes. On April 2, 2020, PMR was negatively associated with community risk (slope −0.16, 95% CI −0.19 to −0.13; *P*<.001). At peak divergence, high-risk ZIP codes reduced mobility by 12 to 13 percentage points less than low-risk ZIP codes (95% CI 11 to 15). Greater PMR was associated with lower subsequent hospitalization risk (incidence rate ratio [IRR] 0.41, 95% CI 0.31 to 0.53; *P*<.001), whereas higher community risk was associated with increased hospitalization risk (IRR 3.03, 95% CI 2.57 to 3.58; *P*<.001). The protective association of mobility reduction was attenuated in higher-risk communities (interaction IRR 2.79, 95% CI 2.08 to 3.73; *P*<.001).

**Conclusions:**

Mobility data can support real-time surveillance of behavioral responses during epidemics. Within-city differences in mobility reduction were associated with subsequent hospitalization risk, but their protective association was weaker in higher-risk communities. Integrating digital mobility data with community risk metrics may improve targeted and equitable public health responses.

## Introduction

As COVID-19 emerged in early 2020, communities worldwide enacted strict lockdowns to curb transmission and ease pressure on health care systems [1-6]. In the United States, policy responses varied substantially by state. Although federal guidelines promoted social distancing [7-11], mask mandates [12,13], and the closure of nonessential businesses [14,15], states differed widely in their implementation, timing, and enforcement [16]. Texas, for example, issued stay-at-home orders on March 20, 2020 [17,18], later than many states, exempted the construction industry from lockdown [19], and began reopening relatively early (May 1, 2020). Within the state, several major cities imposed their own stay-at-home orders days before statewide action and, in some cases, maintained stricter measures. Later in the pandemic, statewide policies, such as limits on elective procedures and business activities, were adaptively implemented based on local hospital and intensive care unit capacity [20].

Across the country, these uneven and evolving policies meant that communities faced different rules and risks depending on where they lived. Yet even where restrictions were strict, not all populations could adhere to them equally [21-33]. Economic instability, job insecurity, and high employment in essential industries limited many individuals’ ability to stay home [19,20,34]. Low-wage workers, particularly in construction [19], agriculture, retail, and food services, often continued working in person without paid sick leave, facing difficult trade-offs between protecting their health and maintaining economic stability for themselves and their families [8,19,21,30,33].

Understanding how these social and economic constraints shaped local behaviors is critical for interpreting the uneven impacts of COVID-19 across communities. Individual-level mobility data, made available early in the pandemic by platforms such as SafeGraph [35], Google [36], and Apple [37], provided critical insights into community movement patterns and improved outbreak forecasts [38]. These anonymized datasets track daily time spent at home and visits to key locations, including workplaces, schools, grocery stores, and medical facilities. However, mobility is only a partial proxy for transmission risk: reductions in movement do not necessarily correspond to proportional reductions in contact, since exposure also depends on factors such as workplace contact intensity and household crowding that mobility metrics do not directly capture.

Studies linking mobility with health care data have shown that low-income populations generally experienced higher mobility, more limited access to health care (including testing and vaccines), and greater COVID-19 disease burden [39-45]. However, most prior research has focused on variation across states or counties, even though substantial variation occurs at finer geographic scales.

Here, we leverage high-resolution mobile device data as a form of digital epidemiological surveillance to characterize behavioral responses during the early COVID-19 pandemic at the ZIP code level. Focusing on the 20 most populous counties in Texas, the second most populous US state, characterized by geographic diversity and decentralized policymaking, we analyze mobility patterns across 623 ZIP codes covering 72% of the state’s population. Using precautionary mobility reduction (PMR) as a proxy for behavioral response, we assess how community-level risk shapes within-city variation in mobility, examine differences in destination patterns, and evaluate associations between mobility and subsequent COVID-19 hospitalization risk. By integrating digital mobility data with socioeconomic and health outcomes, we also assess the potential of these data streams to support real-time public health surveillance and inform targeted responses during emerging epidemics.

## Methods

### Study Design and Population

We conducted an ecological, longitudinal study of mobility patterns and COVID-19 hospitalizations among residents of ZIP Code Tabulation Areas (ZCTAs) in the 20 most populous counties in Texas during February to July 2020. The primary units of analysis were ZCTAs and ZCTA-weeks. Mobility analyses included 623 ZCTAs, representing approximately 72% of the Texas population. COVID-19 hospitalization analyses included 620 ZCTAs for which weekly hospitalization data were available from the Texas Department of State Health Services (DSHS). The study integrated 3 population-level data sources: anonymized mobile device location data, ZCTA-level community risk measures derived from social vulnerability indicators, and weekly COVID-19 hospitalization records.

### Data Collection

#### Mobility Data

We obtained anonymized cell phone location data from SafeGraph, a data provider that aggregates device-level visits from census block groups (CBGs) to specific points of interest (POIs) from numerous mobile apps [35]. To protect user privacy, SafeGraph excludes data if fewer than 2 devices from a given CBG visit a POI in a month. The dataset is not randomly sampled, as inclusion depends on device users having at least 1 location-sharing mobile app. Prior validation studies report that SafeGraph data do not systematically overrepresent CBGs by racial composition, income, or educational attainment at the aggregate county level [44,46,47]. However, more granular audits find that Hispanic, lower-income (<US$ 50,000), and less-educated populations are consistently underrepresented relative to White, higher-income (>US$ 100,000), and college-educated populations, with underrepresentation more pronounced in the South and intensifying after March 2020 [48], overlapping with this study’s geography and study period. We address the implications of this bias for our findings in the *Limitations*. SafeGraph defines each device’s home location as its most frequent nighttime CBG over the preceding 6 weeks.

For our analysis, CBGs were linked to ZCTAs using the Housing and Urban Development-United States Postal Service ZIP Code Crosswalk File [49], which allocates geography based on residential address distributions. ZCTAs were subsequently linked to counties using the corresponding Housing and Urban Development ZIP code–county crosswalk files. Because ZIP codes, ZCTAs, and county boundaries do not perfectly align, minor boundary misclassification is possible; however, the address-based crosswalk approach reduces this risk relative to centroid-based assignments [49]. Hereafter, we refer to ZCTAs as ZIP codes. Visit counts were scaled to account for ZIP codes’ 2020 population [50] and the total number of tracked devices, enabling spatial and temporal comparisons. Hereafter, we refer to these scaled visit counts as visits. Our analysis focused on mobility patterns within the 20 most populous counties in Texas from February 3 to May 31, 2020, relative to the average daily visits during a baseline period (January 6 to February 2, 2020). The baseline period began January 6, 2020, the first Monday after the New Year’s holiday and winter school break, so that mobility patterns had returned to typical, nonholiday levels. It spanned 4 complete weeks to align with our weekly aggregation of visits, and the study period began February 3, 2020, the Monday immediately following the baseline period. Mobility is measured as visits from home locations to POIs regardless of their location within the 20 most populous counties in Texas; that is, POIs could be in the home or away ZIP code (Table S1 in Multimedia Appendix 1).

ZCTAs were included in the mobility analysis if a valid PMR was available for more than 320 of the 333 (96%) days spanning the baseline and study periods. This criterion excluded 98 of the 716 (14%) ZCTAs located in the 20 most populous counties, consistent with SafeGraph’s device-count suppression threshold, which disproportionately affects low-population areas. Excluded ZCTAs did not differ from included ZCTAs in community risk score or hospitalization risk but had significantly smaller populations (Multimedia Appendix 1, Section 5).

For each day during the study period, we defined the PMR, which is calculated as follows:


$${\text{PMR}}_{i,t}=\frac{{\overset{-}{B}}_{i}-V_{i,t}}{{\overset{-}{B}}_{i}+V_{i,t}},$$

where *V*_*i,t*_ are the daily visits from home in ZIP code *i* to a POI regardless of the ZIP code of destination (POI can be located in the same or a different ZIP code from the home ZIP code) on day *t*, and ${\overset{-}{B}}_{i}$ is the baseline daily visits from the home ZIP code i to POIs in any ZIP code for the corresponding day of the week, averaged across the baseline period (January 6 to February 2, 2020). PMR is defined in the range [−1 to 1], where if PMR_*i,t*_>0, ZIP code *i* had ${\bar{B}}_{i}>V_{i,t}$ on day *t* (reduction in visits with respect to the baseline); if PMR_*i,t*_>0, ZIP code *i* had ${\bar{B}}_{i}<V_{i,t}$ on day *t* (increase in visits with respect to the baseline); if PMR_*i,t*_>0, ZIP code *i* had ${\bar{B}}_{i}=V_{i,t}$ on day *t* (no change in visits with respect to the baseline). PMR captures aggregate change in visitation. A change in PMR does not necessarily reflect a personal choice to be cautious. It can also result from other constraints, such as whether a job can be done remotely rather than requiring in-person attendance, whether someone must rely on public transit, or whether someone lacks access to delivery services to meet daily needs. We therefore interpret PMR as a proxy for a broad range of behavioral responses to the pandemic, rather than a simple measure of individual risk perception or compliance.

Additionally, we classified POIs by industry using their North American Industry Classification System codes provided by SafeGraph [51]. This classification included 62 general industry categories, covering more than 4 million unique POIs (Table S2 in Multimedia Appendix 1). We collected detailed information on visit origin and destination ZIP codes. To account for changes in visits to POIs, we calculated the PMR*_i,t,p_*, which is the PMR for POI *p* on day (or week) *t* from home ZIP code *i.* Visits and baseline values were aggregated for each POI separately.

#### Community-Level Risk Score

We used the US Centers for Disease Control and Prevention (CDC)’s Social Vulnerability Index (SVI), a composite measure derived from 15 American Community Survey (ACS) variables designed to estimate a community’s ability to withstand environmental, biological, and other stressors [52]. SVI values are provided at the census tract and county levels as percentile ranks (range 0.0-1.0). For example, an SVI of 0.8 indicates that a community is more vulnerable than 80% of other communities within the comparison group. We calculated the SVI for each ZIP code in Texas using data from the 2016 to 2020 ACS 5-year estimates [53]. We assigned ZIP codes to counties using the R package zipcodeR [54] and recreated the CDC/Agency for Toxic Substances and Disease Registry SVI methodology [55] at the ZIP code scale [56]. Percentile ranks were calculated within Texas rather than nationally, so that a ZIP code’s SVI reflects its relative vulnerability among Texas communities specifically, using all 15 ACS variables underlying the CDC/Agency for Toxic Substances and Disease Registry methodology. To assess sensitivity to the reference population used for ranking, we additionally recalculated the community risk score locally within each of the 20 counties, ranking ZIP codes only against others in the same county rather than statewide; this comparison and its results are reported in *Results*.

SVI could not be computed for 124 of 1935 (6%) Texas ZCTAs, a limitation concentrated in very low-population areas with unstable ACS estimates. Within the 20 most populous counties, this excluded 30 of 716 (4%) ZCTAs from all downstream analyses, since a valid SVI value was required for inclusion in both the mobility and hospitalization datasets (Multimedia Appendix 1, Section 5).

To assess the relationship between social vulnerability and early COVID-19 mobility, measured by the PMR, we calculated the community risk score for each ZIP code. When needed, we grouped ZIP codes into 4 quartiles of equal size—each containing the same number of ZIP codes, from the lowest-risk quartile to the highest-risk quartile—and calculated the mean PMR within each quartile.

#### COVID-19 Health Care Data

We analyzed daily COVID-19 inpatient hospitalization data at the ZIP code level obtained from the Texas DSHS inpatient research data file (institutional review board [IRB] number 23-013). We filtered millions of inpatient records for the state to 65,474 hospital admissions among residents of 620 ZCTAs across Texas during February to July 2020. Patients were categorized as having COVID-19 if the *International Classification of Diseases, 10th Revision* code U07.1 was present on admission as the principal diagnosis or listed among the top 3 comorbidities. These counts reflect inpatient hospitalization events, not unique individuals, in which each record corresponds to 1 inpatient stay, and patients hospitalized more than once during the study period are counted once per admission rather than deduplicated to a single individual [57]. Hospitalizations were aggregated by ZCTA of patient residence and epidemiological week. Patient ZIP codes provided by DSHS were translated to ZCTAs using 2022 Census Topologically Integrated Geographic Encoding and Referencing geographic boundaries for ZCTAs and March 2023 boundaries for ZIP codes from the 2024 Health Resources and Services Administration ZIP Code to ZCTA Crosswalk [58]. Patients originating outside Texas were removed.

These 620 ZCTAs correspond to 620 of the 623 (99.5%) ZCTAs included in the mobility analysis; the remaining 3 (76544, 78234, and 78419) reported no hospitalizations during the study period, 2 of which are predominantly military ZIP codes (Fort Cavazos and Fort Sam Houston), where residents likely receive care through facilities not captured in the DSHS civilian dataset. We also compared hospitalization risk between ZCTAs included in and excluded from the mobility analysis (see *Mobility Data*); the population-normalized hospitalization rate did not differ significantly between the 2 groups (Multimedia Appendix 1, Section 5).

### Statistical Analysis

#### Modeling Longitudinal Mobility Patterns at the ZIP-Code and County-Level

To examine how PMR changed across Texas ZIP codes and to quantify how its association with community-level risk factors changed over time, we fitted a longitudinal generalized additive mixed model (GAMM) with nested random effects. This approach accommodates repeated daily observations within ZIP codes, allows flexible nonlinear temporal trends, and accounts for hierarchical geographic structure by including both county- and ZIP code–level random intercepts. Because mobility behavior is temporally autocorrelated, residuals were modeled using an autoregressive AR(1) process.

Formally, for each ZIP code *i* in county *j* and day *t*, we modeled the centered 7-day rolling mean of the PMR as *i* in county *j* and day *t*, we modeled the centered 7-day rolling mean of the PMR as


$${\text{PMR}}_{i,j,t}=\beta_{0}+f_{1}\left(t\right)+f_{2}\left(t\right){\text{RiskScore}}_{i,j}+c_{j}+z_{i\left(j\right)}+\epsilon_{i,j,t},$$

where *f*_1_ (*t*) is the smooth statewide temporal trend, *f*_2_ (*t*) is a smooth, time-varying coefficient capturing how the PMR-risk association evolved over time, $c_{j}∼\text{N}\left(0,\sigma_{\text{county}}^{2}\right)$ is a county-level random intercept, $z_{i\left(j\right)}∼\text{N}\left(0,\sigma_{\text{ZIP}}^{2}\right)$ is a ZIP code-specific random intercept nested within county *j*, and *∈*_*i,j,t*_ follows an AR(1) structure, $\epsilon_{i,j,t}=\rho \epsilon_{i,j,t-1}+\eta_{i,j,t},\;\eta_{i,j,t}∼\text{N}\left(0,\sigma^{2}\right)$. Under this formulation *f*_2_ (*t*) represents the time-specific association between community-risk and PMR, with *f*_2_(*t*)>0 indicating greater mobility reductions in high-risk ZIP codes at time *t*, and *f*_2_(*t*)>0 indicating smaller mobility reductions among high-risk ZIP codes. All smoothness penalties and variance components, including the AR(1) correlation parameter, were estimated using restricted maximum likelihood (REML).

#### Modeling Structural and Behavioral Drivers of COVID-19 Hospitalizations

We modeled weekly ZIP code–level COVID-19 hospitalizations using a GAMM to account for overdispersed count outcomes, nonlinear epidemic dynamics, and hierarchical spatial structure. The unit of analysis was a ZIP code–week during February to July 2020.

Let *Y*_*i,c,t*_ denote the number of COVID-19 hospitalizations among residents of ZIP code *i*, located in county *c*, during week *t*. We assumed *Y*_*i,c,t*_ denote the number of COVID-19 hospitalizations among residents of ZIP code *i*, located in county *c*, during week *t*. We assumed


$$Y_{i,c,t}∼\text{NegBin}\left(\mu_{i,c,t},\theta \right),$$

where *μ*_*i,c,t*_ is the expected number of hospitalizations and is the dispersion parameter. The conditional mean was modeled on a log scale as


$$\log\left(\mu_{i,c,t}\right)=\log\left(N_{i}\right)+\beta_{0}+\beta_{1}{\text{RiskScore}}_{i}+\beta_{2}{\text{PMR}}_{i,t-4}+\beta_{3}\left({\text{RiskScore}}_{i}\times {\text{PMR}}_{i,t-4}\right)+f\left(t\right)+u_{i}+v_{c},$$

where *N*_*i*_ is the population of ZIP code *i* (included as a logarithmic offset), ensuring inference on hospitalization rates rather than counts. The fixed-effects coefficients are *β*_0_, the baseline log hospitalization rate for a ZIP code with average characteristics at the reference point; *β*_1_, quantifies the association between community risk score and hospitalization risk, capturing structural differences in baseline susceptibility across ZIP codes; and *β*_2_, quantifies the association between 4-week lagged PMR and hospitalization risk to reflect delays between behavioral change, infection, and severe disease. The interaction term *β*_3_ allows the association between PMR and hospitalization risk to vary as a function of the community risk score, reflecting the hypothesis that the epidemiological benefits of mobility reduction are conditioned by underlying socioeconomic constraints.

The term *f*(*t*) is a smooth, nonlinear function of time (weeks since February 10, 2020), modeled using a thin-plate regression spline. This term captures the underlying epidemic trajectory, including rapid early growth and subsequent stabilization, and prevents time-varying epidemic dynamics from being spuriously attributed to PMR or the community risk score.

To account for unobserved spatial heterogeneity, we included random intercepts


$$u_{i}∼\text{N}\left(0,\sigma_{\text{ZIP}}^{2}\right),\;v_{c}∼\text{N}\left(0,\sigma_{\text{county}}^{2}\right),$$

where *u*_*i*_ and *v*_*c*_ capture ZIP code and county-level differences, respectively. Models were estimated using REML with a log link. Exponentiated parametric coefficients are interpreted as incidence rate ratios, representing multiplicative changes in hospitalization rates per unit change in each predictor, conditional on the epidemic trajectory and spatial random effects. For interaction models, marginal effects of PMR were evaluated at representative levels of community risk to facilitate interpretation.

For visualization, marginal model predictions were evaluated at the median study week, averaging over a 3-week window (median ±1 week) to reduce stochastic variation. Observed hospitalization rates were summarized over the same window and, for display only, averaged within quantile-based bins of PMR; binning did not affect model estimation.

All analyses were conducted in R (version 4.5.2; R Foundation for Statistical Computing). GAMMs were fitted using the *mgcv* package (version 1.9-3) with REML for smoothing parameter estimation [59]. Data manipulation and visualization were performed using the tidyverse package (version 2.0.0) [60]. Spatial data processing was conducted using the sf package (version 1.0-21) [61].

### Ethical Considerations

This study was conducted under the Texas DSHS IRB protocol *Spatiotemporal Dynamics of SARS-CoV-2, Influenza, and Influenza-like Illness Across Texas* (DSHS IRB protocol number 23-013). The protocol included a waiver of informed consent because the study involved secondary analysis of existing hospital discharge records and did not involve direct enrollment of participants. COVID-19 hospitalization data were obtained from DSHS under a data use agreement. Mobility data were obtained through Dewey Data (now Advan Patterns), which distributes research versions of the mobility datasets originally produced by SafeGraph. Both datasets were analyzed in aggregated, deidentified form, and no personally identifiable information or individual-level location trajectories were available to the investigators. No attempts were made to identify individual participants. Data were stored and analyzed in secure institutional computing environments in accordance with DSHS data protection requirements and institutional policies. Participants received no compensation.

## Results

In Texas, across the 623 ZIP codes within the 20 most populous counties, the mean PMR across counties increased from −0.0035 (SD 0.13) prior to stay-at-home orders (February 3 to March 15, 2020) to 0.57 (SD 0.11) during the first week of April 2020 (April 6-12, 2020), corresponding to approximately a 73% reduction in mobility relative to baseline (Figure 1A). Notably, a marked decline in mobility occurred shortly after the first confirmed COVID-19 cases were reported in Texas (blue dashed line, Figure 1A), well before the statewide stay-at-home order (gray dashed line, Figure 1A).

**Figure 1.**
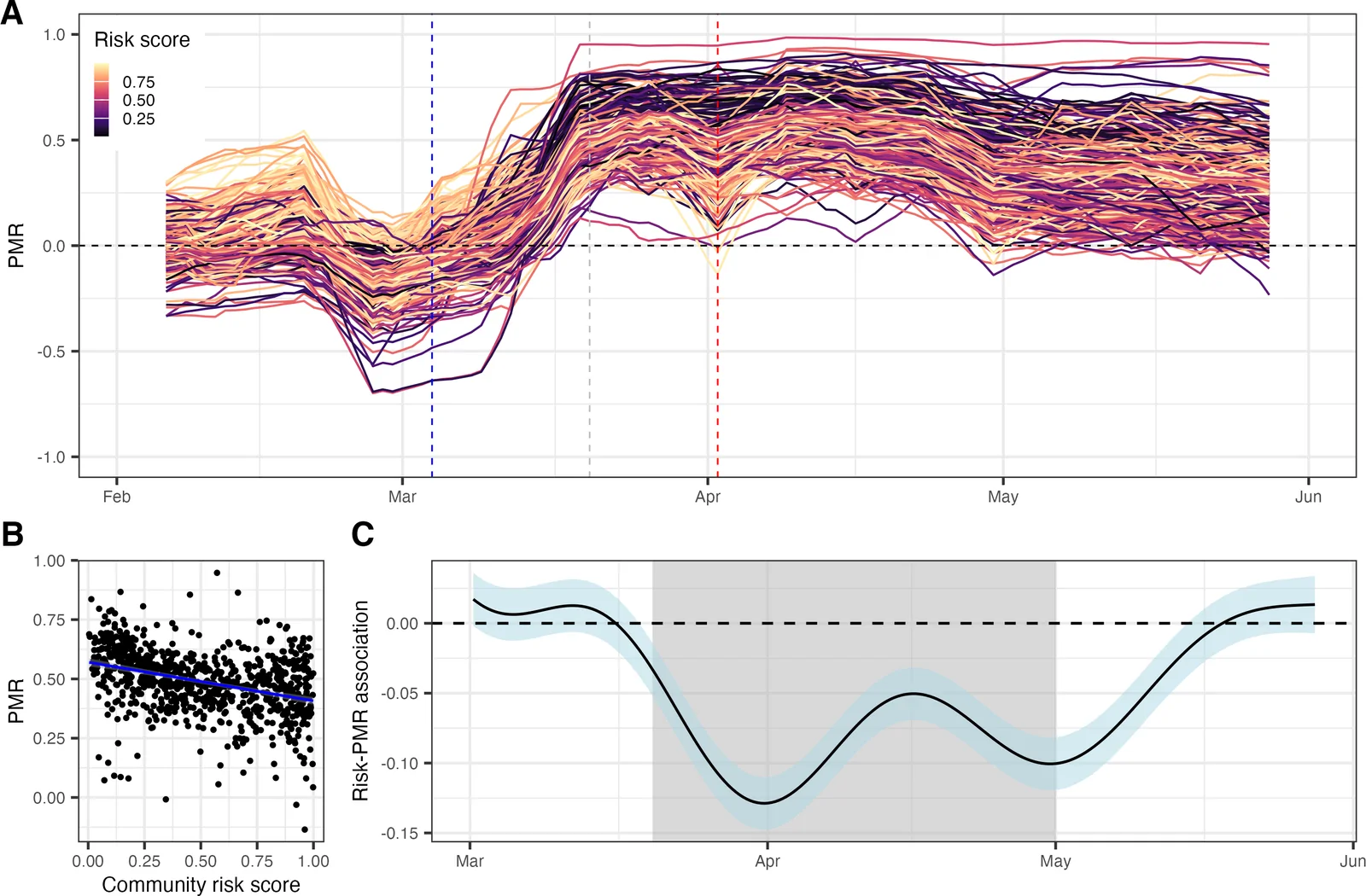
Changes in mobility during the early COVID-19 lockdowns across the 20 most populous counties in Texas. (A) Daily 7-day rolling mean of PMR for 623 ZIP codes from February 5 to May 31, 2020. Lines represent ZIP codes and are colored by community risk score, from lowest risk (dark purple) to highest risk (yellow). The horizontal dashed line indicates PMR=0 (baseline mobility). Vertical dashed lines denote the first reported COVID-19 case in Texas (March 4; blue), the start of the statewide stay-at-home orders (March 20; gray), and April 2, 2020 (red). (B) Association between PMR and community risk score across ZIP codes on April 2, 2020. Points represent ZIP codes; the line shows the fitted linear association (slope −0.16, 95% CI −0.19 to −0.13; *P*<.001). (C) Estimated time-varying association between community risk score and PMR from the longitudinal model. The curve shows the estimated association with a 95% confidence band. The gray shaded region indicates the statewide stay-at-home period (March 20-May 1). PMR: precautionary mobility reduction.

On April 2 (red vertical line, Figure 1A), ZIP codes with lower community risk exhibited larger reductions in mobility than higher-risk ZIP codes (*R*^2^=0.13, *P*<2×10^−16^; Figure 1B). Across the full study period, we fitted a GAMM to the 70,329 ZIP code–day observations of the centered 7-day PMR slope, including smooth functions of time and a time-varying community risk effect, as well as random intercepts for counties and ZIP codes with AR(1) residual correlation (Figure 1C). The model achieved excellent fit (adjusted *R*^2^=0.898, deviance explained=89.9%). The overall temporal trend in PMR was highly nonlinear (time smooth *f*_1_ (*t*): effective degrees of freedom [edf]=9, *F*=2.06×10^4^, *P*<2×10^4^, *P*<2×10^−16^) capturing pronounced changes in mobility during early March followed by a gradual relaxation through May.

Differences in mobility reduction between higher- and lower-risk ZIP codes evolved substantially over time, becoming more pronounced during the early lockdown period (Figure 1C); edf=10, *F*=525, *P*<2×10^−16^. Early in the study period (early March), mobility reductions were similar across ZIP codes regardless of community risk *f*_2_(*t*)≈0. In contrast, during late March through late April, coinciding with the statewide stay-at-home order (March 20-May 1), ZIP codes with lower risk scores began to exhibit significantly greater mobility reductions than higher-risk ZIP codes. At the peak of this disparity in late March, ZIP codes at the 75th percentile of the risk score reduced mobility by approximately 12 to 13 percentage points less than those at the 25th percentile (95% CI 11-15). By late May, differences in mobility reduction by community risk had largely converged. Overall, these patterns indicate a reversal over time in the relationship between social vulnerability and mobility reduction.

County- and ZIP code–level random effects captured substantial residual spatial heterogeneity (county effect *s* (county): edf=17, *F*=4.24×10^4^, *P*<2×10^−16^; ZIP code effect *s* [ZIP]: edf=593.4, *F*=122, *P*=.04), indicating that meaningful variation in mobility persisted beyond that explained by community-level risk factors and statewide temporal trends. Full model specifications and diagnostics are provided in Multimedia Appendix 1, Section 2.

Across counties, overall mobility reductions were pronounced, but the magnitude and timing of disparities by community risk varied (Figure 2 and Figures S2 and S3 in Multimedia Appendix 1). To evaluate county-level heterogeneity, we recalculated the community risk score locally, scaling ZIP codes within each county so that it reflects relative risk within each county rather than statewide ranking. Several large counties, including Harris, Dallas, Travis, and Denton, exhibited time-varying effects that closely mirrored the statewide pattern (Figure 2A). In these counties, the estimated time-varying effect $f_{2}(t)$ crossed into negative values more abruptly than in the statewide model, indicating earlier and stronger divergence in mobility reductions between higher- and lower-risk ZIP codes. Other counties also showed significant associations, but in many cases, the estimated effects were small or the CIs were wide, indicating weaker or more uncertain differences in mobility (Figure S3 in Multimedia Appendix 1). Among them, Travis County showed the earliest divergences (on March 12, 2020), with higher-risk ZIP codes exhibiting smaller mobility reductions. Spatially, higher-risk ZIP codes east of the I-35 corridor (Figure 2B) corresponded to smaller reductions in mobility during early April (Figure 2C), highlighting marked within-county heterogeneity (additional counties shown in Figure S4 in Multimedia Appendix 1).

**Figure 2.**
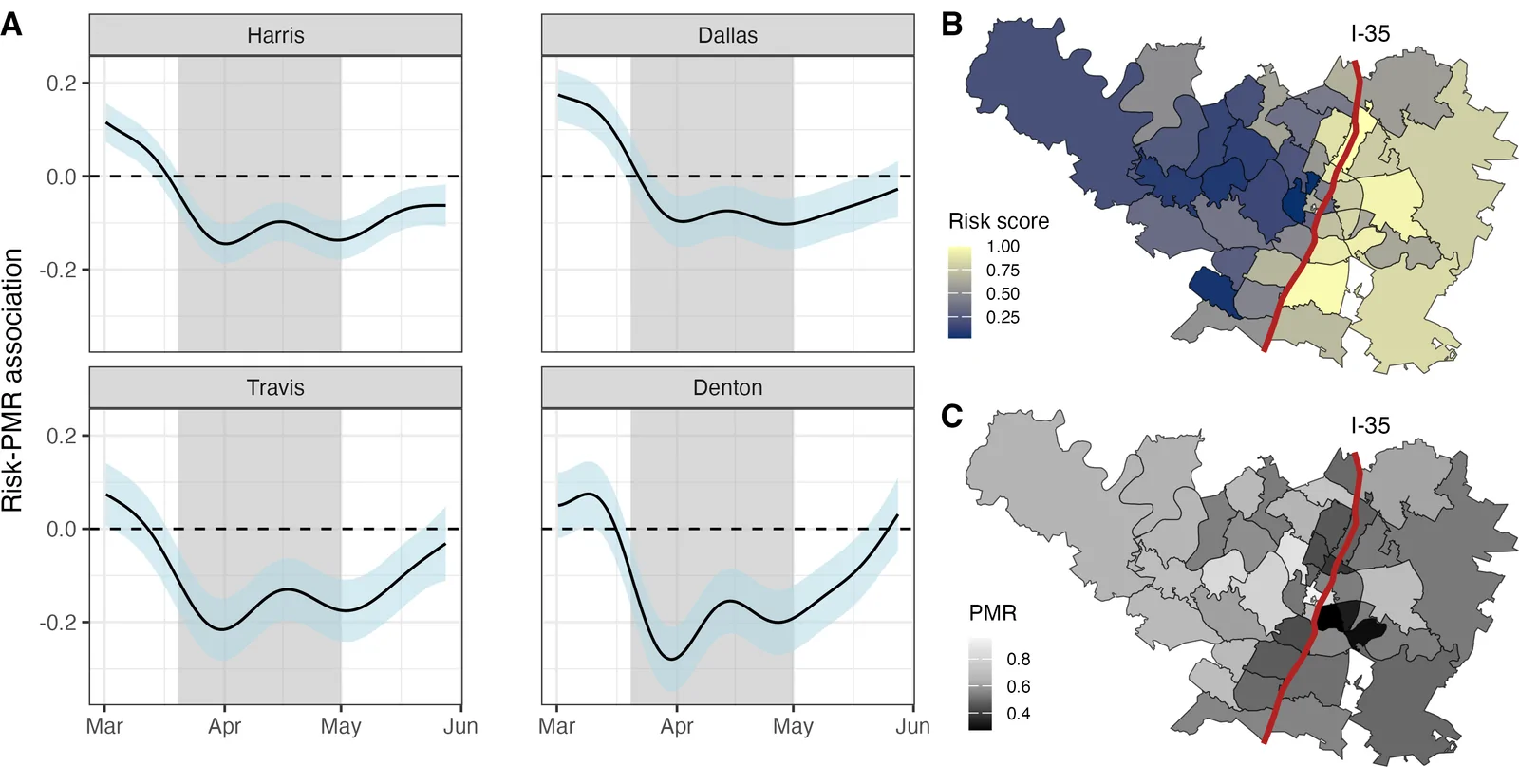
Temporal and spatial patterns of community risk and mobility reduction during the early COVID-19 pandemic in Texas counties. (A) Estimated time-varying association between the community risk score and 7-day PMR in 4 Texas counties (Harris, Dallas, Travis, and Denton). Lines represent model estimates with 95% CIs. Negative values indicate periods when higher-risk ZIP codes exhibited smaller mobility reductions. The shaded region denotes the statewide stay-at-home order (March 20 to May 1, 2020). (B) Community risk levels in Travis County ZIP codes. (C) Mean PMR by ZIP code in Travis County during the early pandemic period (March 30 to April 5). The I-35 corridor is overlaid in panels B and C to illustrate the east-west spatial gradient in community risk and mobility reduction. PMR: precautionary mobility reduction.

Using a negative binomial GAMM of weekly ZIP code–level COVID-19 hospitalizations (February-July 2020), we found that 4-week lagged PMR was significantly associated with lower subsequent hospitalization rates after adjustment for the community risk score (Table 1). Results were robust to alternative lag specifications (full model specifications and diagnostics are provided in Multimedia Appendix 1, Section 3). The model reproduced the observed temporal trajectories and between-ZIP code variability in hospitalization rates (Figure 3A).

**Table 1.** Negative binomial generalized additive mixed model of weekly COVID-19 hospitalizations across Texas ZIP codes (February to July 2020)^a^.

Predictor	β (log rate) (95% CI)	SE	*P* value
Community risk	1.110 (0.945 to 1.274)	0.084	<.001
PMR^b^ (lag 4 wk)	−0.894 (−1.155 to −0.634)	0.133	<.001
PMR × community risk	1.025 (0.734 to 1.316)	0.148	<.001
Intercept	−10.901 (−11.104 to −10.699)	0.103	<.001

a Negative binomial model with a log link and population offset, including PMR (lag 4 wk), community risk score, their interaction, a smooth function of week, and ZIP code– and county-level random intercepts. Model fit: n=10,525 ZIP-weeks; deviance explained=68.3%; θ=5.21; effective degrees of freedom: *s*(WeekIndex)=8.88, ZIP code=415.10, county=17.40. Model diagnostics are provided in Multimedia Appendix 1, Section 3.

b PMR: precautionary mobility reduction.

**Figure 3.**
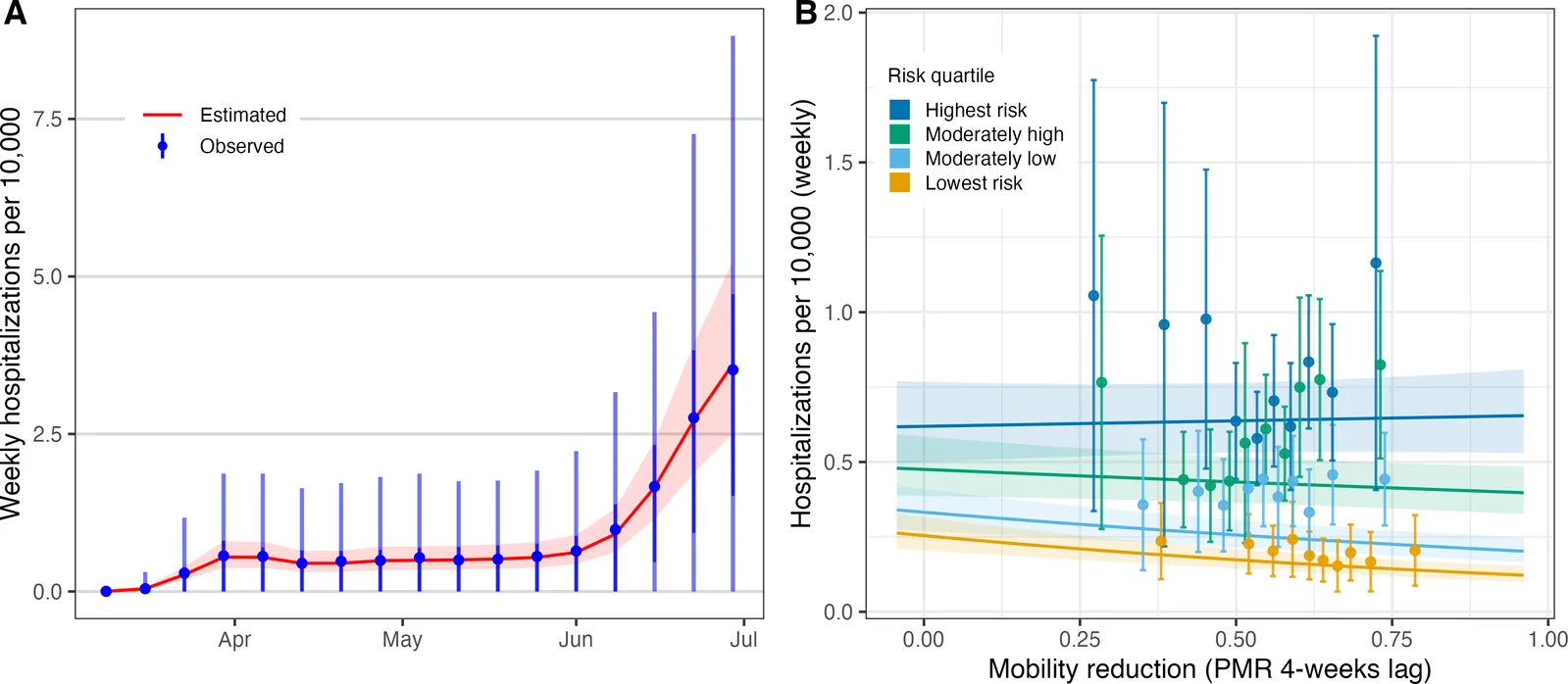
Observed and model-estimated COVID-19 hospitalization patterns across Texas ZIP codes. (A) Weekly COVID-19 hospitalization rates per 10,000 people across Texas ZIP codes, February to July 2020. Blue points represent the observed mean across ZIP codes; vertical bars indicate the IQR (thick blue) and 5th to 95th percentiles (thin blue). The red line and shaded ribbon show model-estimated mean hospitalization rates with 95% CIs. (B) Observed (points with 95% CIs) and model-estimated (lines with 95% confidence ribbons) association between 4-week lagged PMR and weekly COVID-19 hospitalization rates per 10,000 people, stratified by ZIP code community risk score quartile. PMR: precautionary mobility reduction.

The model closely reproduced the observed temporal evolution and distribution of hospitalization rates across ZIP codes (Figure 3A), capturing both the statewide epidemic trajectory and the substantial between-ZIP code variability over time. Greater PMR was associated with lower subsequent hospitalization rates overall (*P*<.001), while higher-risk ZIP codes experienced substantially higher hospitalization burdens independent of mobility. The significant PMR × community risk interaction indicated that the protective association of mobility reduction was strongest in lower-risk ZIP codes and attenuated in higher-risk communities (Figure 3B). Thus, although mobility reductions were associated with reduced hospitalization rates, their epidemiological benefits were not evenly distributed across communities. Model comparison analyses indicated that these findings were robust to alternative specifications, including changes in the complexity of the temporal smooth and the inclusion of county-level random effects (ΔAkaike information criterion≤57). In contrast, removing ZIP code–level random effects or the temporal smooth term resulted in substantial deterioration in model fit (ΔAkaike information criterion=1194 and 7933, respectively), underscoring the essential role of fine-scale geographic heterogeneity and temporal dynamics in explaining hospitalization patterns (Multimedia Appendix 1, Section 3).

The types of destinations visited shifted markedly during stay-at-home orders and differed substantially between ZIP codes in the lowest- and highest-risk score quartiles (Figure 4). Of 62 POI categories, 21 showed significant differences in PMR between the 2 groups for visits within residents’ home ZIP codes (Figure 4, left; POI definitions in Multimedia Appendix 1, Section 4). In contrast, mobility reductions to POIs located outside the home ZIP code were largely similar across risk levels (Figure 4, right).

**Figure 4.**
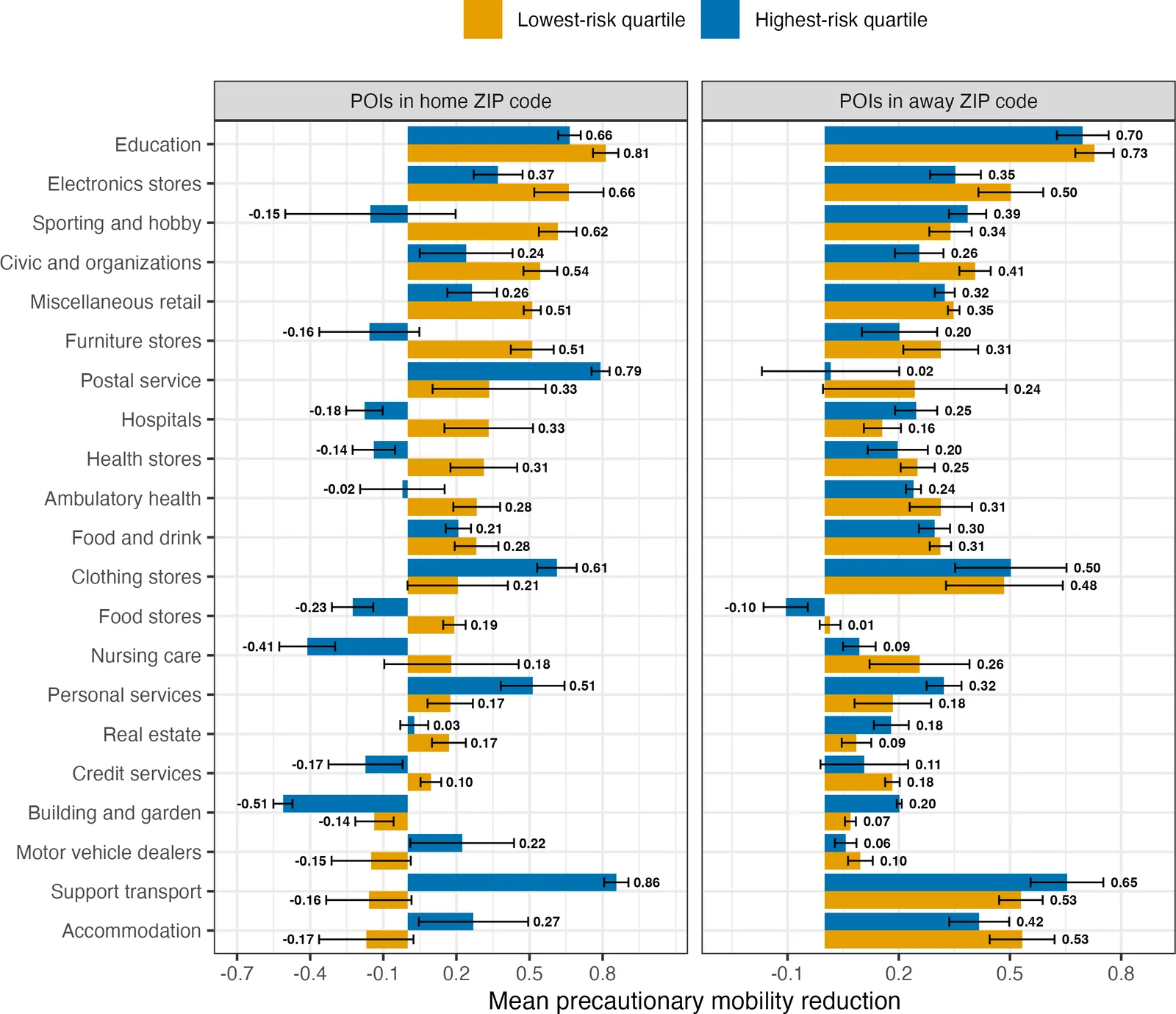
Mobility reduction by community risk and destination type during the initial COVID-19 lockdown in Texas (March 20 to April 13, 2020). Bars show the mean precautionary mobility reduction across economic sectors, comparing ZIP codes in the lowest (orange) and highest (blue) quartiles of the community risk score. Positive PMR values indicate reduced visits relative to the prepandemic baseline. Panels distinguish visits to points of interest (POIs) located within the home ZIP code (left) versus outside the home ZIP code (right). Error bars indicate 95% CIs. Definitions of POI categories are provided in Multimedia Appendix 1, Section 4. Figure S7 shows analogous patterns for February 2020, prior to the implementation of COVID-19 restrictions.

Across most sectors, lower-risk ZIP codes experienced larger mobility reductions, whereas higher-risk ZIP codes often showed smaller—or, in some cases, negative—PMR values, particularly for health-related destinations such as hospitals, ambulatory care facilities, pharmacies, and nursing facilities. For travel outside the home ZIP code, mobility reductions were generally similar across groups, with the notable exception of food-related venues (eg, supermarkets), where the higher-risk ZIP codes exhibited increased mobility relative to baseline.

## Discussion

### Principal Findings

We integrated high-resolution mobility data, community-level risk measures, and ZIP code–level COVID-19 hospitalization records to examine how behavioral responses and structural conditions shaped early COVID-19 outcomes across Texas communities. We found that higher-risk communities reduced mobility less during the period of greatest restrictions and experienced substantially higher subsequent hospitalization rates. Although greater PMR was associated with lower hospitalization risk overall, this protective association was significantly weaker in higher-risk communities. These findings support the value of high-resolution mobility data for understanding within-city heterogeneity and informing public health surveillance.

### Interpretation of Findings

ZIP code–level analyses revealed marked heterogeneity within counties, with neighboring communities exhibiting divergent mobility patterns and hospitalization rates. Such fine-scale variation is largely obscured in county- or state-level analyses and highlights the value of high-resolution data for public health surveillance. Reliance on coarser geographic indicators may therefore limit the ability to identify vulnerable populations and to target interventions effectively within metropolitan areas.

Mobility reductions during the early pandemic were strongly shaped by community constraints. While mobility declined rapidly following the implementation of statewide stay-at-home orders, higher-risk ZIP codes experienced smaller and less sustained reductions during the period of greatest restrictions, and in subgroups directly engaged in newly surged essential-service demand, such as delivery and grocery workers, mobility may have increased rather than merely declined less, consistent with the persistently elevated food-related mobility we observed in these ZIP codes (Figure 4). This divergence likely reflects structural constraints—including essential employment, limited access to remote work, crowded housing, and caregiving responsibilities—rather than differences in awareness, risk perception, or willingness to comply with public health guidance. Consistent with prior research, our findings suggest that the impact of behavior-based interventions depends on underlying socioeconomic conditions and that framing mobility reductions as individual “compliance” risks overlooking the material constraints that shape exposure and mobility [40-43,45,62,63].

Community risk remained a dominant determinant of hospitalization rates throughout the study period. After adjusting for epidemic timing, population size, spatial clustering, and mobility behavior, higher-risk ZIP codes experienced persistently elevated hospitalization rates. Although mobility reduction was associated with lower subsequent hospitalization rates overall, its protective association was attenuated in higher-risk communities, suggesting that similar behavioral changes may yield unequal epidemiological benefits depending on underlying conditions. Mobility reductions alone were insufficient to offset elevated hospitalization risks in high-risk ZIP codes. Sector-specific analyses further highlight the limits of mobility restriction policies. Higher-risk ZIP codes maintained greater mobility to essential destinations, including food, health care, and service-related locations, highlighting the limits of mobility-based interventions in the absence of broader social protections [34,40-42,62,64,65]. As a result, population-wide restrictions may reduce transmission on average while leaving higher-risk communities disproportionately exposed to severe disease outcomes [19,33,40,44,66,67].

From a public health perspective, these findings highlight the importance of context-sensitive epidemic control strategies [68]. Interventions that rely primarily on behavioral modification may inadvertently reinforce disparities if differential capacity to comply is not addressed [67-70]. Integrating community risk metrics with real-time mobility and surveillance data could help identify communities that require targeted support, including paid sick leave, workplace protections, access to testing, and timely health care services. Such measures may help ensure that mitigation strategies are both effective overall and more equitable in their impact across communities.

### Limitations

This study has several strengths, including its fine spatial resolution, integration of multiple data sources, and use of flexible modeling approaches to capture nonlinear epidemic dynamics and hierarchical geographic structure. Nonetheless, limitations should be acknowledged. Mobility data are not derived from a random sample. While aggregate validation studies report no systematic overrepresentation by race, income, or education at the CBG level [44,46,47], finer-grained audits find that low-income, Hispanic, and less-educated individuals are underrepresented relative to their higher-income, White, and college-educated counterparts, and that this gap widens in the South and after March 2020 [48]. This raises the possibility that our mobility measures do not fully capture behavior in the most vulnerable ZIP codes in our sample. Mobility metrics capture movement patterns but not other protective behaviors, such as mask use or physical distancing, nor activity redistributed to the home, such as remote work, telehealth, or grocery and food delivery replacing in-person shopping. Even after accounting for these unmeasured behaviors, mobility reduction is not equivalent to contact or transmission-risk reduction. Two ZIP codes with identical PMR values can carry different exposure risks if, for example, one has more in-person, close-contact jobs or more crowded housing. Associations between PMR and hospitalization risk should therefore be read as reflecting these unmeasured behavioral and structural differences across communities, not as a claim that reduced mobility directly causes fewer hospitalizations by itself. Hospitalization data may reflect differences in health care access, admission, and reporting practices, or underlying health status across communities.

County-level random intercepts in our statistical model partially absorb systematic differences in reporting and admission practices that operate at the county level, though residual ZIP code–level heterogeneity in health care access could not be directly modeled with the data available. For these factors to fully account for the observed association between community risk and hospitalization rates, they would need to vary systematically with community risk itself; while we cannot rule out such a pattern, we are not aware of data supporting it. Future work incorporating direct measures of health care access, such as hospital bed capacity, insurance coverage, or testing rates, could test this formally through sensitivity analyses. Although our models include ZIP code– and county-level random intercepts, these capture hierarchical grouping rather than explicit spatial correlation, and neighboring ZIP codes are not statistically independent given cross-boundary commuting, shared workplaces, and shared health care facilities. We tested this directly: Moran *I* on ZIP code–level residuals from the mobility model showed significant positive spatial autocorrelation (*I*=0.19, *P*<.001; Multimedia Appendix 1, Section 2). Because community risk itself follows a known geographic gradient, this residual spatial structure means that standard errors assuming independent observations likely understate true uncertainty, and we cannot rule out that it is partly correlated with community risk itself.

In addition, because our analyses were conducted at the ZIP code level, associations observed at the community level may not reflect individual-level relationships. Finally, the analyses were restricted to the early phase of the COVID-19 pandemic in Texas, and associations may differ under later variants, vaccination campaigns, evolving testing practices, or changing public health policies.

### Conclusions

High-resolution mobility data provide a valuable complement to traditional surveillance systems by capturing population-level behavioral responses in near real time. However, our findings indicate that behavioral indicators should be interpreted alongside measures of underlying community vulnerability because identical behavioral changes may not translate into equivalent health benefits across populations. Integrating mobility data with community risk metrics may therefore improve the targeting of interventions during future epidemics while helping public health agencies identify communities requiring additional structural support.

## Supplementary material

10.2196/97405 Multimedia Appendix 1 Complementary analysis.

## Abbreviations

ACS: American Community Survey
CBG: census block group
CDC: US Centers for Disease Control and Prevention
DSHS: Department of State Health Services
edf: effective degrees of freedom
GAMM: generalized additive mixed model
IRB: institutional review board
IRR: incidence rate ratio
PMR: precautionary mobility reduction
POI: points of interest
REML: restricted maximum likelihood
SVI: Social Vulnerability Index
ZCTA: ZIP Code Tabulation Area
