# Supplementary material for: Community Risk, Mobility Patterns, and COVID-19 Hospitalization Risk in Texas: ZIP Code–Level Ecological Longitudinal Study
Source: JMIR Public Health Surveill. 2026 Nov 4;12:e97405. doi: 10.2196/97405 (PMC13734555; doi:10.2196/97405)
Supplement: Supplementary file 1 [file publichealth-v12-e97405-s001.pdf]

Supplemental material.

Community risk, mobility patterns, and COVID-19 hospitalization risk: A ZIP code–level analysis using mobile-device data

Jose L. Herrera-Diestra[1], Dongah Kim[2], Shraddha Ramdas Bandekar[2], Emily Javan[2], Spencer J. Fox[3], Remy Pasco[4], Lauren Ancel Meyers[2].

1- Department of Mathematics, Tarleton State University
2- Department of Integrative Biology, The University of Texas at Austin
3- School of Informatics, Computing, and Cyber Systems, Northern Arizona University
4- BioFrontiers Institute, University of Colorado at Boulder

Section 1. County level statistics and list of industries considered.

Table S1. Population size, number of ZIP codes and mean community risk score in the 20 Texas counties analyzed.

County	Population	Number of ZIP codes	Mean community risk score
Harris	4,646,630	131	0.586
Dallas	2,606,868	81	0.571
Tarrant	2,049,770	64	0.456
Bexar	1,952,843	68	0.573
Travis	1,226,805	46	0.341
Collin	973,977	25	0.205
Hidalgo	855,176	20	0.917
El Paso	836,062	26	0.727
Denton	833,822	26	0.262
Fort Bend	765,394	17	0.416
Montgomery	571,949	21	0.413
Williamson	547,604	19	0.279
Cameron	421,666	13	0.801
Nueces	361,540	22	0.655
Brazoria	360,677	15	0.450

Bell	348,574	17	0.527
Galveston	332,885	15	0.488
Lubbock	304,808	18	0.553
Webb	273,526	22	0.763
Jefferson	254,340	16	0.540

Table S2: List of general categories of the North American Industry Classification System (NAICS) in Texas.

Electronics and Appliance Stores	Building Material and Garden Equipment and Supplies Dealers	Social Assistance	Transit and Ground Passenger Transportation
Food and Beverage Stores	Health and Personal Care Stores	Personal and Laundry Services	Administration of Economic Programs
Gasoline Stations	Sporting Goods, Hobby, Musical Instrument, and Book Stores	Food Manufacturing	Printing and Related Support Activities
Clothing and Clothing Accessories Stores	Other Information Services	Data Processing, Hosting, and Related Services	Rail Transportation
General Merchandise Stores	Insurance Carriers and Related Activities	Waste Management and Remediation Services	Truck Transportation
Miscellaneous Store Retailers	Hospitals	Securities, Commodity Contracts, and Other Financial Investments and Related Activities	Crop Production
Real Estate	Performing Arts, Spectator Sports, and Related Industries	Rental and Leasing Services	Heavy and Civil Engineering Construction
Professional, Scientific, and Technical Services	Accommodation	Merchant Wholesalers, Nondurable Goods	Construction of Buildings
Educational Services	Repair and Maintenance	Postal Service	Utilities
Ambulatory Health Care Services	Justice, Public Order, and Safety Activities	Beverage and Tobacco Product Manufacturing	Textile Product Mills
Nursing and Residential Care Facilities	Miscellaneous Manufacturing	Electrical Equipment, Appliance, and Component Manufacturing	Nonmetallic Mineral Product Manufacturing
Museums, Historical Sites, and Similar Institutions	Merchant Wholesalers, Durable Goods	Couriers and Messengers	
Amusement, Gambling,	Furniture and Home	Telecommunications	

and Recreation Industries	Furnishings Stores		
Food Services and Drinking Places	Support Activities for Transportation	Apparel Manufacturing	
Religious, Grantmaking, Civic, Professional, and Similar Organizations	Motion Picture and Sound Recording Industries	Fabricated Metal Product Manufacturing	
Specialty Trade Contractors	Credit Intermediation and Related Activities	Administrative and Support Services	
Motor Vehicle and Parts Dealers	Management of Companies and Enterprises	Warehousing and Storage	

Section 2. GAMM model: modeling longitudinal mobility patterns at the ZIP-code and county-level

Table S3. Summary of parametric and smooth-term estimates for the GAMM evaluating PMR dynamics across Texas ZIP codes. Reported are parametric coefficients, smooth-term edf, F-statistics, p-values, and overall model fit, including adjusted R^2 and deviance explained.

**Parametric coefficients - Intercept**				
**estimate**	**std_error**	**t_value**	**p_value**	
0.2882747	0.01053718	27.35785	0	
**Smooth terms**				
**smooth**	**edf**	**ref_df**	**F_value**	**p_value**
s(Date_num)	8.994902	8.999943	20598.3707	0
s(Date_num):RiskScore_State	9.956199	9.999376	524.6630	0
s(County)	17.009496	19.000000	42422.1498	0
s(Zip)	593.416677	621.000000	122.0252	0.03525124
**Overall model fit**				
**Adjusted R^2**	**Deviance explained**	**Scale estimate**	**N obs.**	**fREML**
0.898	0.899	0.00375	70329	-98534

To assess model adequacy, we examined the standard diagnostic panels generated by the gam.check() function in the *mgcv* package. These diagnostics evaluate whether the generalized additive mixed model (GAMM) satisfies key assumptions related to residual behavior, nonlinearity, variance structure, and overall fit.

The quantile–quantile (QQ) plot of deviance residuals showed only mild deviations in the extreme tails, with the majority of residuals lying close to the theoretical normal reference line. This indicates that the Gaussian likelihood is appropriate for the centered 7-day PMR used as the response. The histogram of residuals further confirmed that the residuals are symmetric and tightly centered around zero, consistent with a well-behaved error structure.

Residuals plotted against the linear predictor showed no systematic curvature or strong heteroscedasticity. This suggests that the nonlinear smooth terms included in the model—specifically, the statewide temporal trend and the time-varying effect of SVI—adequately capture the underlying structure of the data. The hierarchical random effects

for counties and ZIP codes help account for baseline geographic differences, and the AR(1) term addresses temporal autocorrelation within each ZIP code's time series.

The plot comparing observed and fitted values revealed a strong correspondence between the two, with only modest dispersion around the mean trend. This visual pattern is consistent with the high overall model performance (adjusted R^2 = 0.898; deviance explained = 89.9%). To assess whether these random effects and the AR(1) term fully account for spatial dependence among ZIP codes, we additionally tested for residual spatial autocorrelation. We computed ZIP-level mean residuals from the fitted model and constructed a queen-contiguity neighbor list from ZCTA polygons for the 20 counties in the analysis. Moran's I indicated significant positive residual spatial autocorrelation ($I=0.191$, $z=7.34$, $p<.001$), meaning residuals from geographically adjacent ZIP codes remained more similar than expected under spatial randomness. This indicates that the county- and ZIP-level random intercepts, while capturing substantial hierarchical variation, do not fully absorb spatial dependence between neighboring communities.

Taken together, these diagnostics confirm that the GAMM with county-level and ZIP-level random effects and AR(1) residual structure provides an appropriate and stable characterization of longitudinal PMR trends and their time-varying association with community-level risk, though residual spatial autocorrelation indicates that neighboring ZIP codes are not fully independent and that standard errors assuming independence may understate true uncertainty.

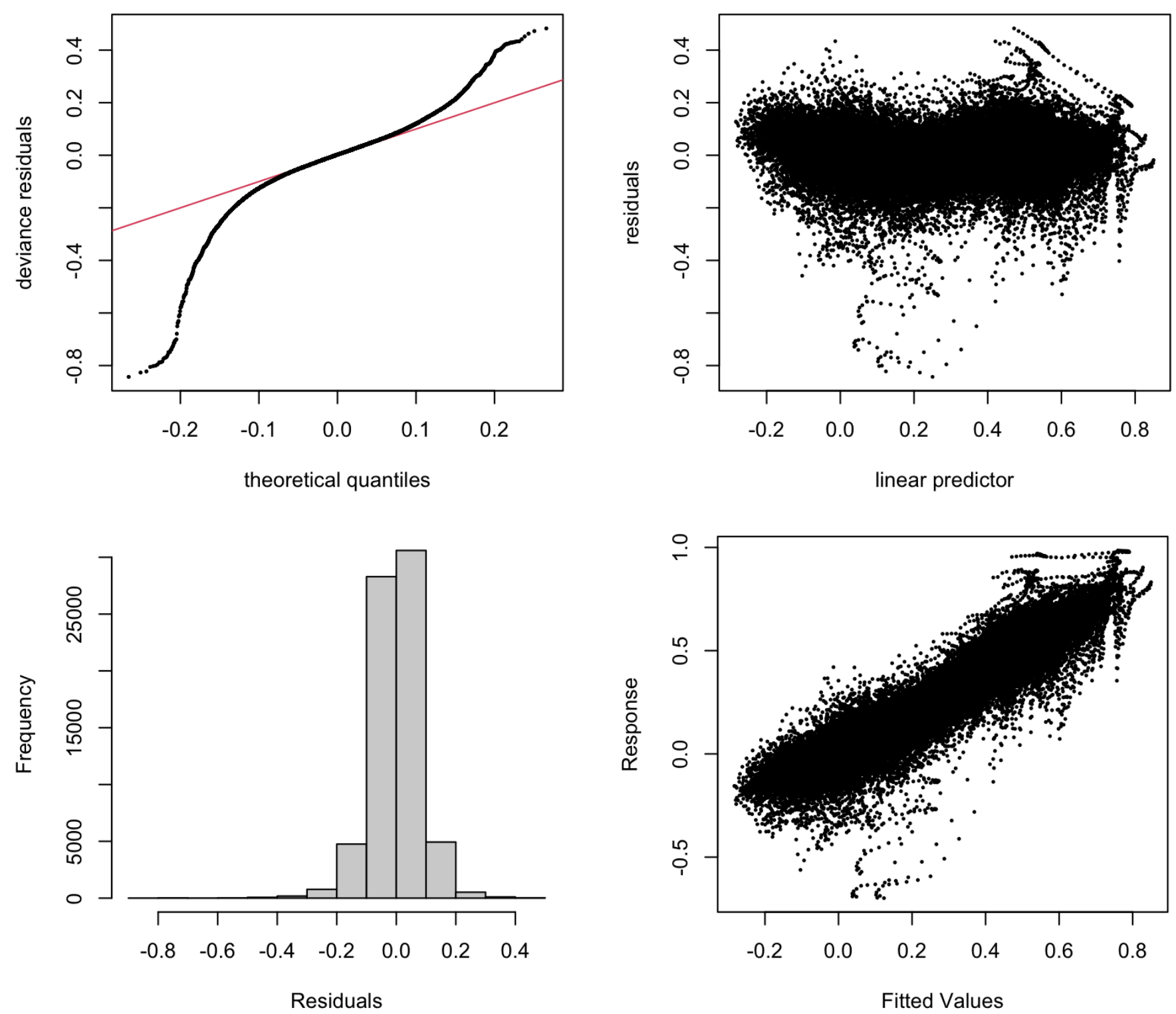

Figure S1. Diagnostic plots for the GAMM, including a QQ-plot, residuals versus fitted values, residual histogram, and observed versus fitted values. The panels show no major deviations from model assumptions, supporting adequate residual behavior and overall model fit.

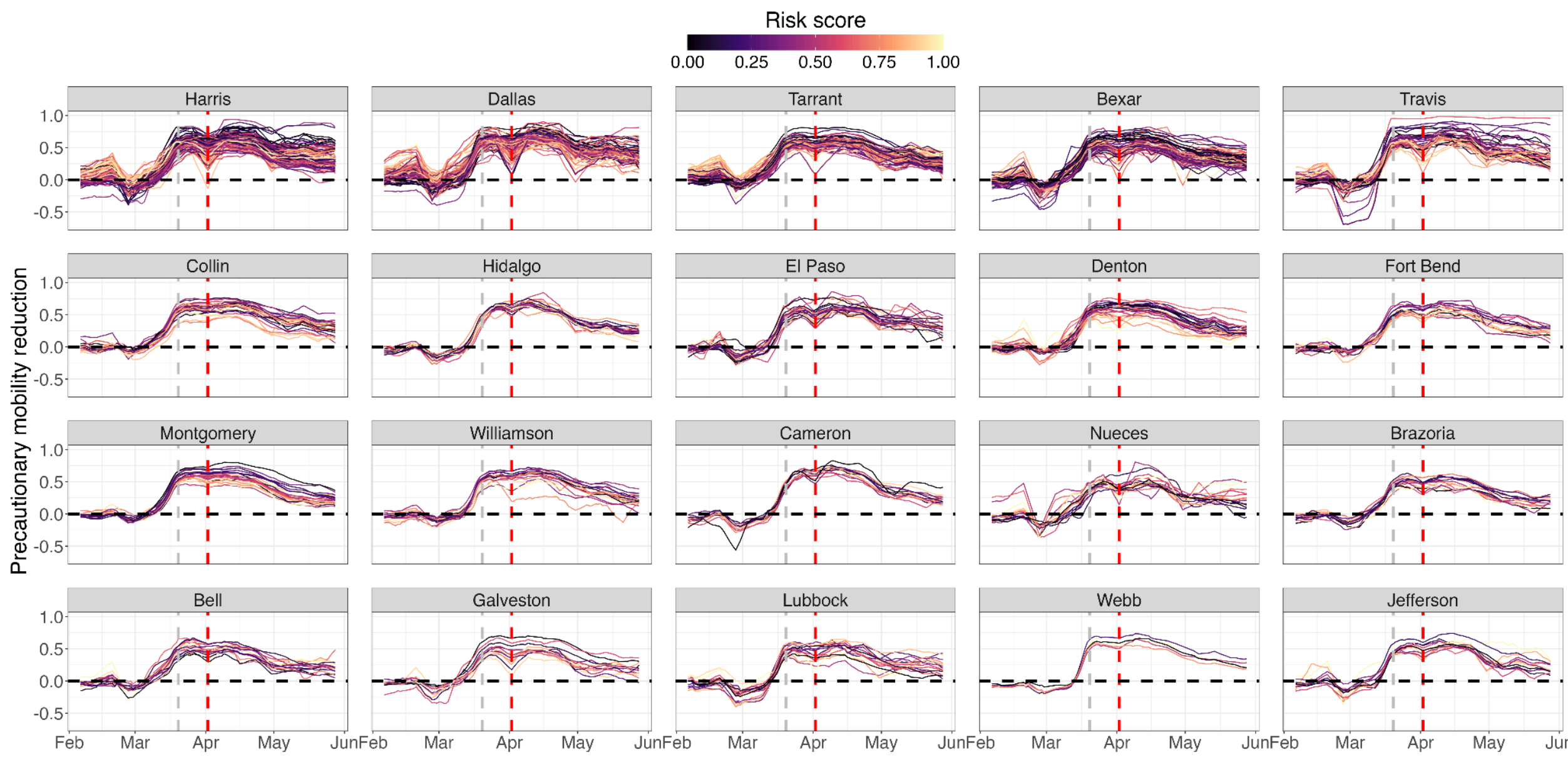

Figure S2. Daily Precautionary Mobility Reduction (PMR) for each ZIP code (lines) in the 20 most populous counties in Texas from February 3 to May 31, 2020. Colors correspond to the community risk score of ZIP codes, from lowest risk (black) to highest risk (yellow). The dashed horizontal line indicates the reference mobility ratio of zero. The vertical dashed gray and red lines correspond to the first day of state-wide lockdown (March 20) and one day on the first week of April (April 2), respectively.

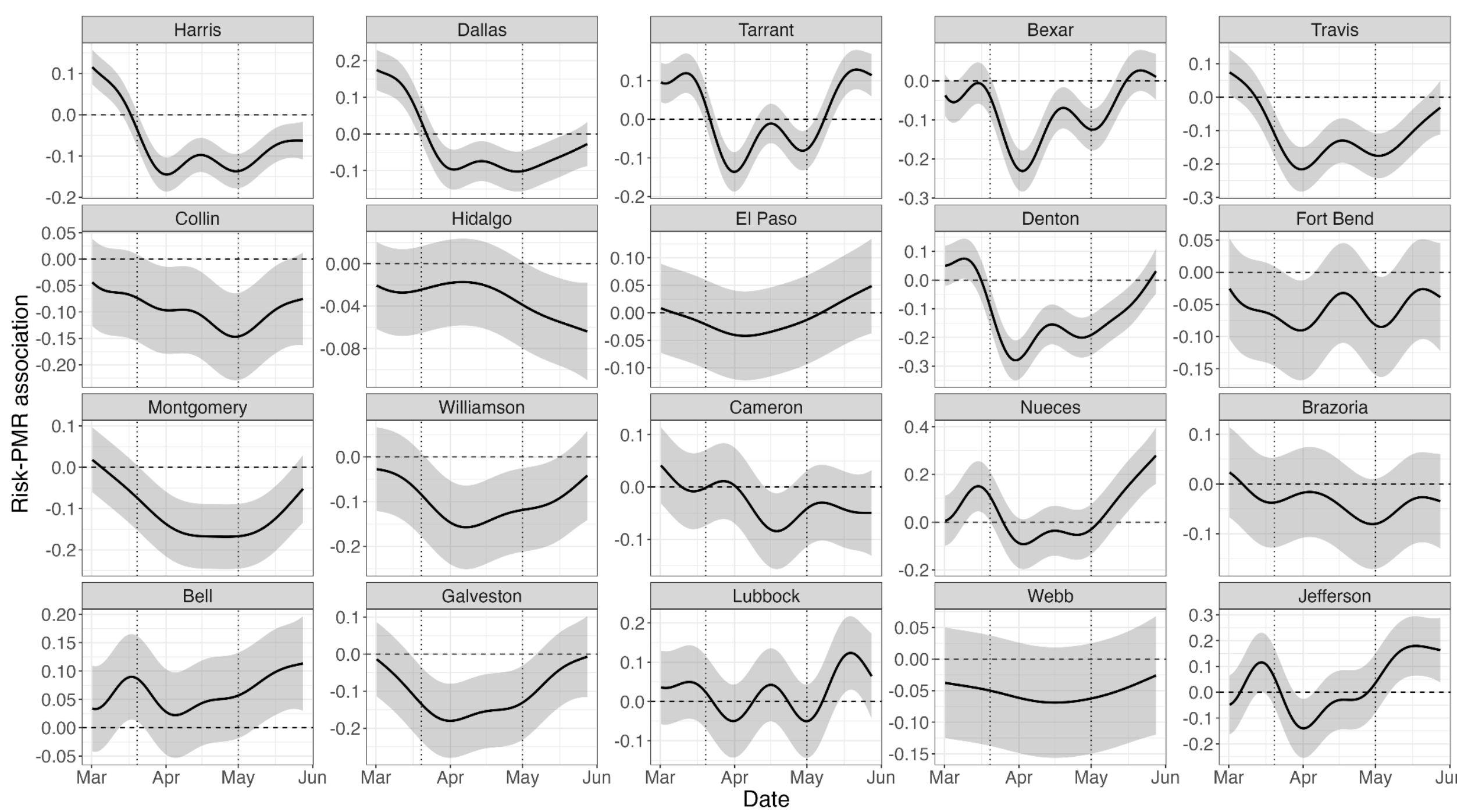

Figure S3. Time-varying effect of *locally scaled* community risk score on the 7-day rolling slope of precautionary mobility reduction (PMR) for the 20 most populous Texas counties. Curves show the estimated smooth effect f_2(t) from county-specific GAMs, with shaded areas representing pointwise 95% confidence intervals. Positive values indicate periods when higher-risk ZIP codes decreased mobility

more strongly, while negative values indicate weaker or delayed reductions in higher-risk ZIP codes. Vertical dotted lines show the Texas statewide stay-at-home order period (March 20–May 1, 2020).

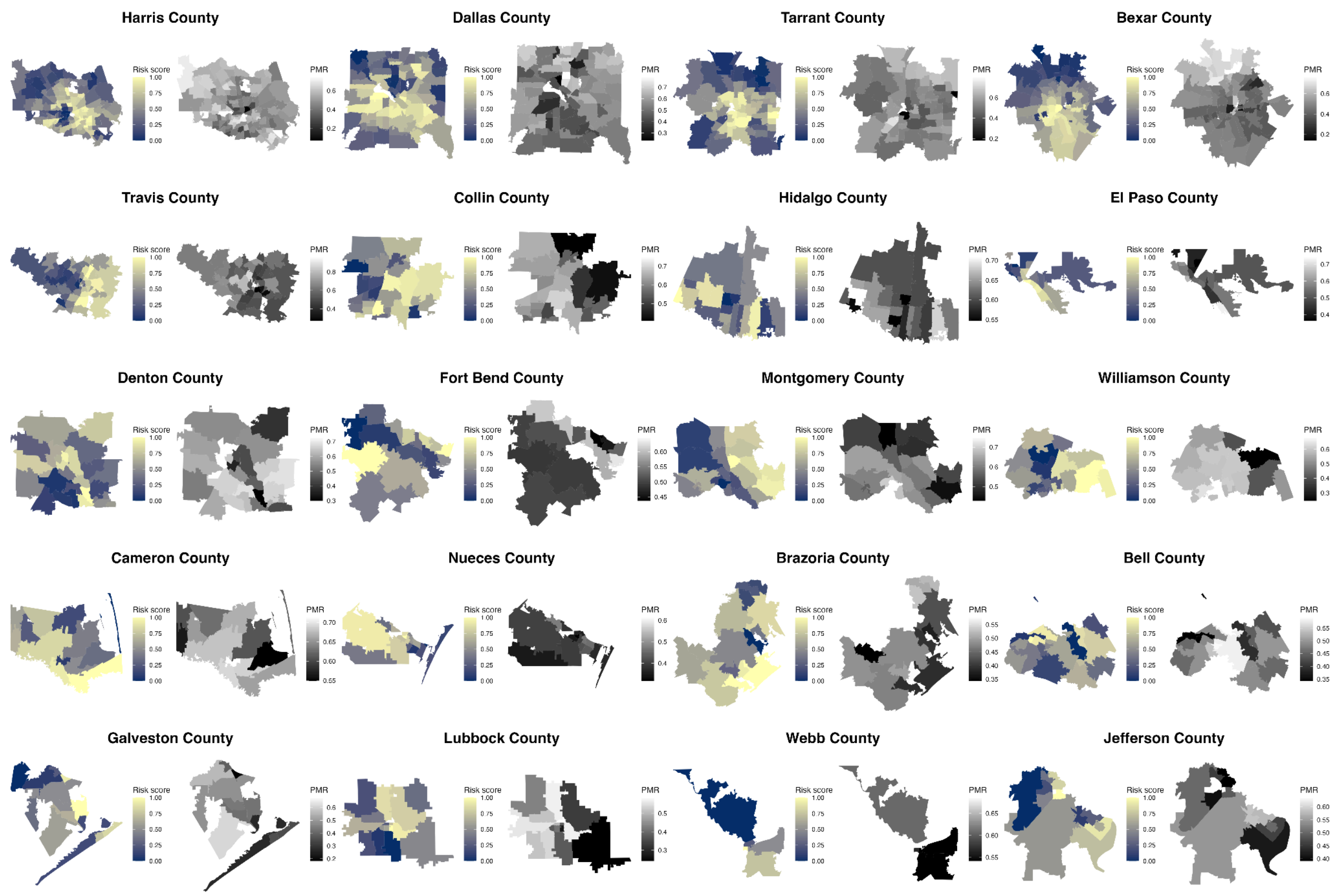

Figure S4. ZIP code–level spatial heterogeneity in community risk score and precautionary mobility reduction across major Texas counties. For each of the 20 most populous Texas counties, maps show the distribution of the community risk score (left panel of each county pair) and the mean Precautionary Mobility Reduction (PMR; right panel of each county pair) at the ZIP Code Tabulation Area (ZCTA) level. PMR is computed as the mean value over March 30–April 5, 2020. Color scales are shown separately for community risk score and PMR to facilitate within-county comparison of spatial patterns.

Section 3. Modeling structural and behavioral drivers of COVID-19 hospitalizations

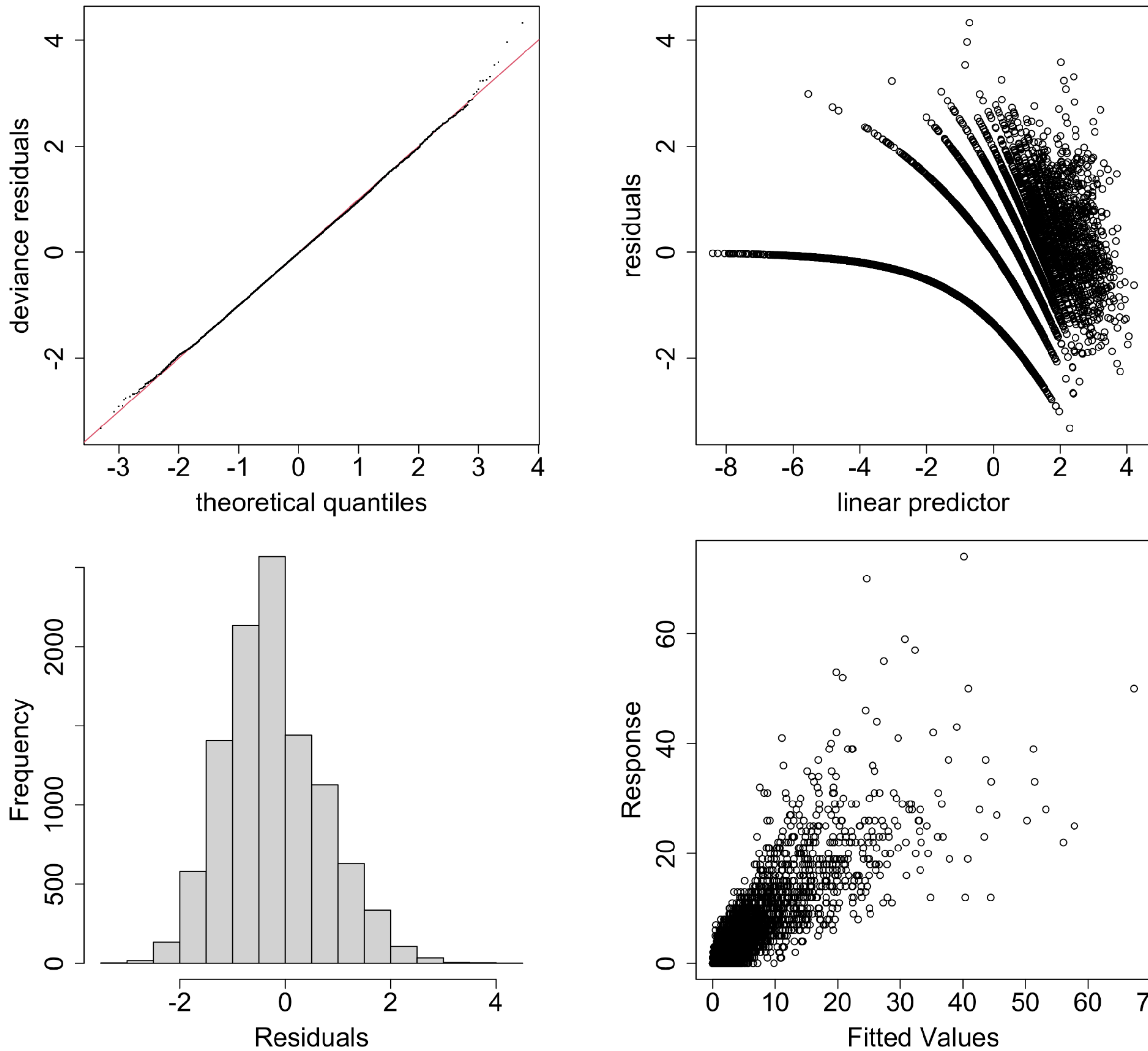

Figure S5. Diagnostic plots for the negative binomial GAMM of weekly COVID-19 hospitalizations. ***Top left:*** Quantile–quantile plot of deviance residuals against theoretical normal quantiles. Residuals closely follow the reference line across most of the distribution, with minor deviations in the extreme upper tail corresponding to localized hospitalization surges during peak epidemic weeks. ***Top right:*** Deviance residuals plotted against the linear predictor. The characteristic fan-shaped pattern reflects the mean–variance relationship of the negative binomial distribution combined with a logarithmic link and population offset, as well as the presence of many ZIP code-weeks with low or zero hospitalizations early in the epidemic. No systematic structure remains after accounting for temporal dynamics and spatial random effects. ***Bottom left:*** Histogram of deviance residuals. Residuals are centered near zero and right-skewed, as expected for overdispersed count data. ***Bottom right:*** Observed hospitalizations versus fitted values. Increasing variability at higher fitted values reflects stochastic epidemic surges and heterogeneous ZIP code-level risk; the model targets the conditional mean hospitalization rate rather than exact peak counts.

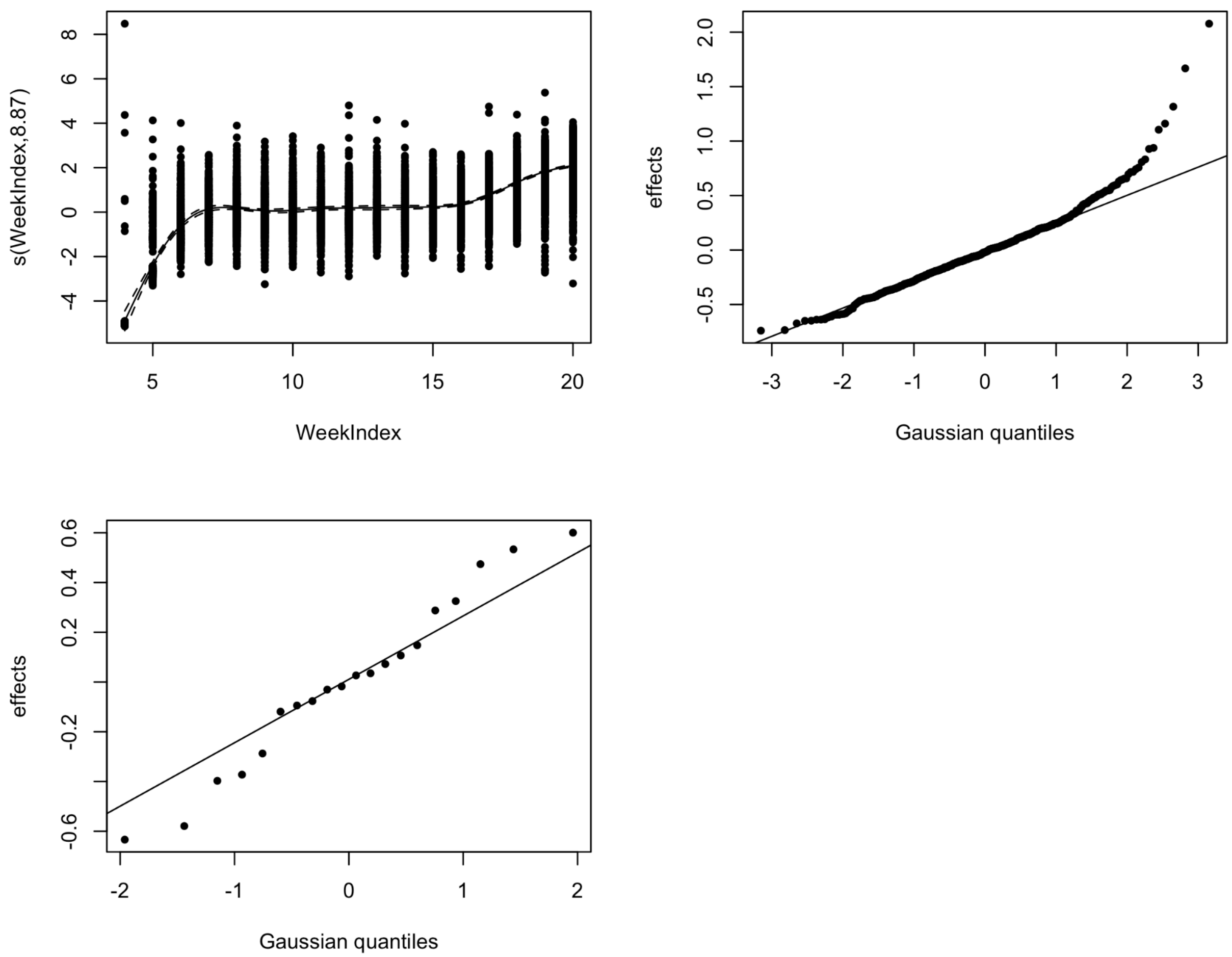

Figure S6. Estimated smooth and random-effect components from the GAMM. ***Top left:*** Estimated smooth function of time, s(WeekIndex), with effective degrees of freedom (edf = 8.58). The smooth captures the nonlinear epidemic trajectory during February–July 2020, including rapid early growth and subsequent stabilization, while preventing time-varying dynamics from being spuriously attributed to mobility or community risk score. ***Top right:*** Quantile–quantile plot of ZIP-code random intercepts, s(PAT_ZCTA) (edf ≈ 413.8). The approximately linear pattern indicates that ZIP-level heterogeneity is well modeled, with modest deviations in the upper tail corresponding to ZIP codes with substantially elevated baseline hospitalization risk. ***Bottom:*** Quantile–quantile plot of county-level random intercepts, s(County) (edf ≈ 17.3). County-level effects are comparatively smaller and closely follow the reference line, indicating limited but systematic spatial heterogeneity beyond ZIP-level variation.

Sensitivity analysis on PMR effects to lag choice

We evaluated lag structures ranging from 0 to 7 weeks to account for the delay between changes in mobility and subsequent hospitalizations. Although later lags yielded lower AIC values, these models were estimated on progressively smaller samples due to lag construction. Lag 4 represented the earliest delay at which the PMR main effect was strongly negative, the interaction between PMR and community risk score was large and highly significant, and all coefficients were stable and statistically robust. This lag also aligns with biologically plausible delays between exposure, infection progression, and hospitalization. Therefore, we selected the 4-week lag as our primary specification.

Table S4. Sensitivity analysis of the association between precautionary mobility reduction (PMR) and weekly COVID-19 hospitalization across alternative lags. Each row corresponds to a separate negative binomial GAMM with identical specification, differing only in the PMR lag. All models include a smooth time term, ZIP code- and county-level random intercepts, and population offset. Positive associations at short lags likely reflect reactive mobility reductions in response to rising epidemic activity, whereas negative associations at longer lags are consistent with delayed protective effects of sustained mobility reduction.

PMR lag (weeks)	β (log rate)	SE	p-value	IRR [95% CI]	Deviance explained
0	1·53	0·160	<0·001	4·62 [3·38, 6·32]	0·758
1	1·24	0·153	<0·001	3·45 [2·55, 4·66]	0·743
2	0·377	0·146	0·01	1·46 [1·10, 1·95]	0·725
3	-0·378	0·140	0·007	0·69 [0·52, 0·91]	0·705
4	-0·89	0·133	<0·001	0·41 [0·31, 0·53]	0·683
5	-1·01	0·126	<0·001	0·36 [0·28, 0·46]	0·657
6	-1·15	0·121	<0·001	0·32 [0·25, 0·41]	0·639
7	-0·985	0·118	<0·001	0·37 [0·29, 0·47]	0·642

Model Comparison (Nested & Nearby Specifications)

To assess the robustness of the primary model, we compared the full generalized additive mixed model to alternative specifications varying the complexity of the temporal smooth, inclusion of interaction terms, and random-effects structure. Model performance was evaluated using ΔAIC relative to the full model and deviance explained.

Changes in the basis dimension of the temporal smooth (week_k15 and week_k8) resulted in minimal changes in model fit (ΔAIC ≤ 30), with nearly identical incidence rate ratios (IRRs) for PMR and the PMR × community risk (SVI) interaction, indicating that the estimated associations were insensitive to smooth complexity. Removing the county-level random effect modestly increased AIC (ΔAIC = 57) without materially altering effect estimates.

In contrast, removing the interaction term (ΔAIC = 41) or PMR main effect (ΔAIC = 95) reduced model fit, supporting their contribution to explaining hospitalization variability. Notably, exclusion of ZIP-level random effects (ΔAIC = 1,194) or the temporal smooth term (ΔAIC = 7,933) resulted in substantial deterioration in model fit, underscoring the essential role of fine-scale geographic heterogeneity and temporal dynamics in capturing hospitalization patterns.

Model	ΔAIC	Deviance Explained	PMR IRR (at SVI=0)	Interaction IRR
Full	0	68.3%	0.41	2.79
week_k15	+4	~68%	~0.40	~2.75
week_k8	+30	~68%	similar	similar
no_county_re	+57	~68%	similar	similar
no_interaction	+41	~68%	—	—
no_pmr	+95	—	—	—
no_zip_re	+1194	—	—	—
no_week_smooth	+7933	—	—	—

Section 4. Detailed description of NAICS codes with significant differences in PMR between lowest-risk and highest-risk subpopulations.

Definitions were extracted from documentation in downloadable files in https://www.census.gov/naics/

441 Motor Vehicle Dealers

The Motor Vehicle and Parts Dealers sector encompasses retail establishments that sell motor vehicles, recreational vehicles, motorcycles, boats, and automotive parts from fixed locations such as showrooms, open lots, or service centers. This category includes a diverse range of Points of Interest (POIs) such as new and used car dealerships(441110, 441120) selling automobiles, SUVs, and vans; recreational vehicle (RV) dealers (441210) offering motor homes and travel trailers; and motorcycle, boat, and other motor vehicle dealers (441222, 441228) selling motorcycles, ATVs, personal watercraft, boats, snowmobiles, and utility trailers. It also includes automotive parts and accessories stores (441310), such as auto supply shops, speed shops, and stores selling stereos, truck caps, and replacement components, as well as tire dealers (441320) specializing in tire and tube sales and installation. Many of these establishments also provide related repair, maintenance, and installation services. In essence, POIs in this sector range from large new car dealerships and RV lots to small auto parts retailers and tire shops, all focused on the retail sale and servicing of motor vehicles and their components.

442 Furniture Stores

The Furniture and Home Furnishings Stores sector includes retail establishments that sell new furniture and decorative home products from showrooms or specialty stores, often offering interior design or installation services. POIs in this category include furniture stores (442110) selling household, office, outdoor, and baby furniture, mattresses, and related items—sometimes alongside appliances or home electronics. It also includes floor covering stores (442210) specializing in carpets, rugs, vinyl, and tile flooring, often providing installation and repair services. The home furnishings group(44229) covers a variety of specialty stores such as window treatment shops (442291) selling curtains, drapes, blinds, and shades, and all other home furnishings stores (442299) offering household décor and utility items like linens, lamps, chinaware, kitchenware, glassware, picture frames, and housewares. Altogether, this sector covers POIs ranging from large furniture showrooms and mattress outlets to small décor, lighting, and flooring boutiques, reflecting a broad mix of home interior and furnishing retail businesses.

443 Electronics Stores

The Electronics and Appliance Stores sector includes retail establishments that sell new household appliances and consumer electronics from showrooms or specialty stores, often with demonstration areas and technical staff. POIs in this sector include household appliance stores (443141) that sell items such as refrigerators, dishwashers, ovens, microwaves, washers, dryers, coffee makers, sewing machines, air conditioners, and vacuum cleaners—sometimes also providing appliance repair services. It also covers electronics stores (443142) specializing

in or offering a broad range of consumer electronics such as televisions, computers, cameras, audio systems, mobile phones and accessories, and home entertainment systems. These establishments may also sell prepackaged computer software and prerecorded media (e.g., CDs, DVDs, games) and often provide technical support, installation, and repair services. Overall, this category captures POIs ranging from large electronics chains and appliance showrooms to smaller specialty retailers focused on gadgets, computing, and home technology.

444 Building & Garden

The Building Material and Garden Equipment and Supplies Dealers sector includes retail establishments that sell materials, tools, and supplies for home improvement, construction, repair, and landscaping, often operating from large indoor/outdoor facilities designed for heavy goods like lumber or garden equipment. POIs in this category include home centers (444110) such as large hardware and improvement stores offering a wide range of materials—lumber, plumbing, electrical goods, paint, tools, hardware, and garden supplies—in departmental layouts. It also covers paint and wallpaper stores (444120) specializing in interior and exterior finishes; hardware stores (444130) offering general tools, fasteners, and builder supplies; and other building material dealers (444190) focusing on specialized materials such as fencing, glass, lumber, plumbing and electrical supplies, kitchen and bath fixtures, prefabricated buildings, and flooring. The sector also includes lawn and garden stores—specifically outdoor power equipment stores (444210) selling and servicing mowers, chainsaws, and trimmers, and nursery, garden center, and farm supply stores (444220) selling plants, seeds, sod, fertilizers, and farm feed. Overall, this subsector covers POIs ranging from large home improvement chains and hardware stores to local lumberyards, garden centers, and farm supply shops, all supporting construction, renovation, and landscape maintenance needs.

445 Food Stores

The Food and Beverage Stores sector includes retail establishments that sell food, beverages, and related products for off-premise consumption, typically operating from fixed locations equipped with refrigeration and food-handling facilities. POIs in this category range from large supermarkets and grocery stores (445110) offering a full line of food products—fresh produce, meats, seafood, dairy, baked goods, and packaged foods—to smaller convenience stores(445120) that carry limited essentials like milk, snacks, and drinks. It also includes specialty food stores (4452), such as meat markets (445210), fish and seafood markets (445220), fruit and vegetable markets (445230), and other specialty shops (44529) that sell items like baked goods, confections, coffee and tea, spices, nuts, and gourmet products (e.g., 445291–445299). Finally, beer, wine, and liquor stores (445310) retail packaged alcoholic beverages for home consumption. Overall, this subsector covers POIs such as supermarkets, local grocers, corner stores, butcher and seafood markets, bakeries, specialty delis, and liquor shops, all focused on providing food and drink products to households rather than prepared meals for immediate consumption.

446 Health Stores

The Health and Personal Care Stores sector includes retail establishments that sell health-related products, personal care goods, and wellness items from fixed point-of-sale

locations, often staffed by trained professionals such as pharmacists, opticians, or beauty consultants. POIs in this category include pharmacies and drug stores (446110), which sell prescription and over-the-counter medications, health aids, and basic personal care items; cosmetics, beauty supplies, and perfume stores (446120), focusing on skincare, haircare, makeup, grooming, and fragrances; and optical goods stores (446130), which sell and fit prescription eyeglasses, contact lenses, and nonprescription eyewear. The category also includes other health and personal care stores (44619), such as health supplement shops (446191) offering vitamins, nutritional and body-enhancement products, and medical supply or assistive device retailers (446199) that sell prosthetics, hearing aids, convalescent equipment, and sickroom supplies. Overall, this subsector covers POIs ranging from large chain drugstores and optical centers to independent vitamin shops and beauty boutiques, serving a broad spectrum of health, wellness, and personal care needs.

448 Clothing Stores

The Clothing and Clothing Accessories Stores sector includes retail establishments that sell new clothing, shoes, jewelry, and related accessories, typically from showrooms, boutiques, or department-style stores where staff provide assistance on style, fit, and fashion trends. POIs in this sector include clothing stores (4481), such as men's (448110), women's (448120), children's and infants' (448130), and family clothing stores (448140), which sell apparel for specific or multiple demographics and often offer basic alteration services. It also includes clothing accessories stores(448150) that sell hats, scarves, belts, handbags, costume jewelry, wigs, and other fashion accessories, as well as specialty clothing stores (448190) such as bridal boutiques, lingerie shops, fur and leather apparel stores, and uniform suppliers. Additionally, shoe stores (448210) retail a wide variety of footwear, from dress shoes and boots to sneakers and sandals. The sector also covers jewelry, luggage, and leather goods stores (4483), including jewelry stores (448310) selling fine jewelry, watches, and silverware—often with repair services—and luggage and leather goods stores (448320) offering luggage, handbags, wallets, belts, gloves, and related accessories. Overall, POIs under this code range from large apparel retailers and department stores to small boutiques, shoe shops, and fine jewelry stores, representing the fashion and personal accessory retail landscape.

451 Sporting & Hobby

The Sporting Goods, Hobby, Musical Instrument, and Book Stores sector includes retail establishments that sell goods and supplies for sports, recreation, hobbies, music, and reading, often staffed by personnel knowledgeable in the use or performance of these products. POIs in this sector include sporting goods stores (451110), which sell athletic and outdoor recreation equipment such as bicycles, camping and fishing gear, fitness and exercise equipment, golf supplies, and specialty footwear. It also includes hobby, toy, and game stores (451120) offering toys, puzzles, board games, model kits, and general craft supplies, and sewing, needlework, and piece goods stores (451130) that sell fabrics, patterns, yarn, sewing machines, and upholstery materials. Musical instruments and supplies stores (451140) sell instruments, sheet music, and related equipment and may provide repair, rental, or instructional services. Additionally, book stores and news dealers (4512) include bookstores (451211) specializing in new books and newsstands (451212) selling newspapers, magazines, and other periodicals.

Altogether, POIs in this subsector range from large chain sporting goods and music stores to independent craft shops, local bookstores, and news kiosks, serving a broad spectrum of leisure, creative, and educational activities.

453 Misc. Retail

The Miscellaneous Store Retailers sector includes a wide variety of specialty retail establishments that sell goods not classified elsewhere, often catering to niche markets, collectibles, or personalized products. POIs in this category include florists (453110) selling cut flowers, floral arrangements, and potted plants; office supplies, stationery, and gift stores (4532) such as office supply shops (453210) retailing stationery, school and office materials, and gift, novelty, and souvenir stores (453220) offering greeting cards, seasonal decorations, and souvenir items. It also includes used merchandise stores (453310), such as thrift shops, antique stores, consignment shops, and used book or clothing stores. The other miscellaneous retailers group (4539) encompasses pet and pet supplies stores (453910) selling live pets, food, and accessories; art dealers (453920) operating galleries and selling original or limited-edition artworks; and manufactured home dealers (453930) retailing new or used mobile homes. The broad “all other” category (45399) includes tobacco stores (453991) selling cigars, cigarettes, and smokers’ supplies, as well as various specialty retailers(453998) such as art supply stores, candle and trophy shops, swimming pool and hot tub dealers, cemetery monument vendors, fireworks stores, and general merchandise auction houses. Altogether, this subsector covers POIs ranging from flower shops, thrift stores, and pet stores to art galleries, cigar shops, and specialty supply retailers, reflecting a diverse mix of niche and lifestyle-oriented retail businesses.

488 Support Transport

The Support Activities for Transportation sector includes establishments that provide specialized services essential to the operation of transportation systems, serving air, rail, water, road, and freight modes as well as general logistics support. These POIs typically do not transport passengers or freight themselves, but instead handle operational, logistical, and maintenance functions.

Key activities include airport operations and air traffic control (488111, 488119), aircraft maintenance, security screening, and testing services (488190); rail transportation support (488210) such as railcar servicing, terminal operation, and loading/unloading; and water transportation support (4883), encompassing port and harbor operations(488310), marine cargo handling (488320), navigational and tugboat services (488330), and ship repair or marine surveying (488390). For road transportation, it includes motor vehicle towing and roadside assistance (488410) and roadway-related services such as toll or bridge operations, pilot car escorting, and truck weighing (488490). Freight transportation arrangement firms (488510) act as freight forwarders, shipping agents, and customs brokers, organizing shipments across carriers and modes. Finally, the other support activities category (4889) covers packing and crating companies (488991), pipeline terminals, stockyards, and carpool/vanpool coordinators (488999).

Altogether, POIs under NAICS 488 include airports, towing services, ports, tugboat operators, rail terminals, logistics brokers, customs agents, and packing firms—the infrastructure and service network that enables the smooth operation of global and domestic transportation systems.

491 Postal Service

The Postal Service sector includes establishments engaged in providing mail collection, sorting, and delivery services under a universal service obligation, typically operated by the national postal authority and its contracted partners. POIs in this category (491110) cover all activities of the U.S. Postal Service (USPS) and its rural or subcontracted post offices, which handle letters, printed materials, and small parcels that can be transported without heavy equipment. These establishments maintain the infrastructure for public mail services, including post offices, mail processing centers, and delivery units, and often offer express or parcel delivery services using the same system. Unlike private couriers (classified under 492), postal service providers operate under legal mandates to ensure nationwide accessibility, affordability, and regular mail delivery. In essence, POIs in this subsector encompass post offices, mail sorting facilities, and rural postal outlets, forming the backbone of the national mail and small-parcel delivery network.

522 Credit Services

The Credit Intermediation and Related Activities sector includes institutions that lend money, issue credit, and facilitate financial transactions. It covers three main groups: depository credit intermediation (5221), including banks, savings institutions, and credit unions that accept deposits and provide loans; nondepository credit intermediation (5222), such as credit card companies, mortgage and consumer lenders, and specialized financing firms; and activities related to credit intermediation (5223), including mortgage brokers, payment processors, and money transfer or check-cashing services. Overall, this sector encompasses banks, credit unions, mortgage companies, credit card issuers, and financial service providers that form the backbone of lending, credit, and payment systems in the economy.

531 Real Estate

The Real Estate sector includes establishments involved in owning, leasing, managing, selling, or valuing properties. It has three main areas: lessors of real estate (5311), which rent or lease residential (531110) and commercial properties (531120), operate self-storage facilities (531130), or lease land and other real estate (531190); real estate agents and brokers (531210), who help clients buy, sell, or rent homes and commercial spaces; and related real estate activities (5313), including property managers (531311–531312), appraisers (531320), and services like escrow or title offices (531390). Altogether, this sector includes landlords, leasing offices, storage facilities, real estate agencies, appraisers, and property managers, forming the foundation of the property market and real estate transactions.

611 Education

The Educational Services sector includes establishments that provide instruction, training, and educational support across all levels and disciplines—from early education to higher learning and professional development. It encompasses academic institutions such as elementary and

secondary schools (611110), junior colleges (611210), and universities and professional schools (611310) granting associate, bachelor's, graduate, or specialized degrees. It also includes vocational and professional training providers (611410–611519), such as business, computer, cosmetology, flight, nursing, or trade schools that offer career-focused education and workforce development programs. Specialized and enrichment schools (611610–611699) provide instruction in areas like fine arts, sports, language, tutoring, and personal development, while educational support services (611710) assist learning through testing, counseling, exchange programs, and curriculum consulting. Together, these institutions—ranging from schools and universities to training centers and educational consultancies—form the foundation of the nation's lifelong learning and skills development system.

621 Ambulatory Health

The Ambulatory Health Care Services covers outpatient medical providers and centers that treat patients without overnight stays. POIs include physician offices (primary care and specialists, plus psychiatry/psychoanalysis) and dental offices; other practitioner clinics such as chiropractors, optometrists, mental health counselors/therapists, physical/occupational/speech therapy and audiology practices, podiatry, midwives, dietitians, acupuncturists, and other licensed practitioners. It also spans outpatient care centers—family planning, mental health & substance-use clinics, HMO medical centers, dialysis centers, freestanding ambulatory surgical centers, urgent care / freestanding emergency centers, and sleep or pain clinics. Diagnostic facilities include medical laboratories (e.g., blood and pathology testing) and imaging centers (MRI, CT, X-ray, ultrasound). Home health agencies provide skilled nursing, therapies, hospice/infusion, and personal care in the home. Other ambulatory services include ambulance (ground/air) bases, blood and organ banks, and miscellaneous screening/monitoring services (e.g., health screenings, hearing tests, fitness evaluations, smoking-cessation, pacemaker monitoring). Overall, expect POIs like clinics, group practices, urgent care and surgery centers, imaging/lab draw sites, home-health agencies, ambulance stations, and blood banks.

622 Hospitals

The Hospitals subsector includes establishments that provide inpatient medical, surgical, and specialized care, integrating physician services, nursing, diagnostics, and continuous patient monitoring along with meals and accommodation. These facilities form the foundation of the inpatient healthcare system, often complemented by outpatient and emergency services. It includes general medical and surgical hospitals (622110) that treat a broad range of illnesses, injuries, and conditions with full diagnostic and surgical capabilities; psychiatric and substance abuse hospitals (622210) offering long-term, supervised treatment for mental health and addiction disorders through medical, psychological, and rehabilitative care; and specialty hospitals (622310) focused on specific medical needs such as rehabilitation, chronic illness, pediatrics, cardiology, or orthopedics. Together, these institutions—ranging from community hospitals to specialized treatment centers—represent the central infrastructure for comprehensive, long-term, and acute medical care in the U.S.

623 Nursing Care

The NAICS 623 – Nursing and Residential Care Facilities subsector includes establishments

that provide long-term housing and care for people who need ongoing medical, personal, or social support. Unlike hospitals, these facilities focus on extended, non-acute care in residential settings. It includes four main groups: nursing care facilities (623110)such as nursing homes, inpatient hospices, and convalescent centers that provide 24-hour skilled nursing and rehabilitation; residential facilities for intellectual or developmental disabilities, mental health, or substance abuse (623210–623220) that offer housing, supervision, and counseling with limited medical care; continuing care and assisted-living communities for the elderly (623311–623312) that combine housing, meals, and personal assistance—with or without on-site nursing care; and other residential care facilities (623990) such as group homes for youth, foster children, or people with disabilities. Overall, this subsector includes nursing homes, assisted-living centers, rehabilitation residences, and group homes, forming the core of long-term and supportive residential care.

721 Accommodation

The Accommodation subsector includes establishments that provide lodging or short-term housing for travelers and temporary residents, ranging from hotels and motels (721110–721120) to bed-and-breakfasts and hostels (721191–721199), as well as RV parks, campgrounds, and vacation camps (721211–721214) for outdoor stays, and rooming houses, dormitories, and workers' camps (721310) offering longer-term or group housing. These facilities may also provide meals, housekeeping, recreation, and event services, serving both leisure and business travelers. Overall, this sector represents the core of the lodging and hospitality industry, supporting tourism, recreation, and temporary residence across diverse accommodation types.

722 Food & Drink

The Food Services and Drinking Places subsector includes establishments that prepare and serve meals, snacks, and beverages for immediate consumption, whether eaten on-site, taken to go, or delivered. It spans a wide variety of businesses—from restaurants, cafés, and coffee shops to caterers, food trucks, and bars—differing in service style, formality, and setting. This subsector covers special food services (7223) such as contract food providers (722310) operating cafeterias in schools or hospitals, caterers (722320) serving events like weddings or conferences, and mobile vendors (722330) such as food trucks and carts. It also includes drinking places (722410) like bars, taverns, and nightclubs that focus on alcoholic beverages with optional entertainment. The largest group, restaurants and other eating places (7225), ranges from full-service restaurants (722511) with table service and menus, to limited-service or fast-food outlets (722513), buffets and cafeterias (722514), and snack and beverage shops (722515) like coffeehouses, ice cream parlors, and juice bars. Collectively, these establishments form the backbone of the prepared food and beverage industry, serving everyday dining, social gatherings, and special occasions across diverse settings.

812 Personal Services

The Personal and Laundry Services subsector includes establishments that provide a wide variety of personal, household, and small-business services (812) focused on individual care and convenience. It covers personal care services (8121) such as barber shops, beauty and nail salons, day spas, tanning and massage parlors, tattoo studios, and weight-loss centers; death

care services (8122) including funeral homes, crematories, and cemeteries; and laundry and dry-cleaning services (8123), from coin-operated laundromats and dry cleaners to uniform and linen suppliers. It also includes other personal services (8129) like pet grooming and boarding, photofinishing labs, parking garages and valet services, wedding planning, bail bonding, dating and escort agencies, and personal fitness training. Overall, this subsector represents the broad range of businesses that provide appearance, hygiene, memorial, and lifestyle support directly to individuals and households.

813 Civic & Organizations

The Religious, Grantmaking, Civic, Professional, and Similar Organizations subsector (813) includes establishments that organize, promote, and support religious, charitable, civic, professional, and membership-based activities. It encompasses religious organizations (8131) such as churches, temples, synagogues, and mosques that administer or promote religious activities; grantmaking and giving services (8132) including charitable foundations, voluntary health organizations, and fundraising entities that support education, science, health, and cultural causes; and social advocacy organizations (8133) that advance social, environmental, human rights, and community causes. It also includes civic and social organizations (8134) such as alumni associations, social clubs, veterans' groups, and fraternal lodges that promote civic or social interests, and business, professional, labor, political, and similar organizations (8139) like trade associations, bar associations, labor unions, and political action committees that represent members' professional, economic, or political interests. Overall, this subsector represents the institutional and organizational fabric of social, cultural, charitable, and professional life.

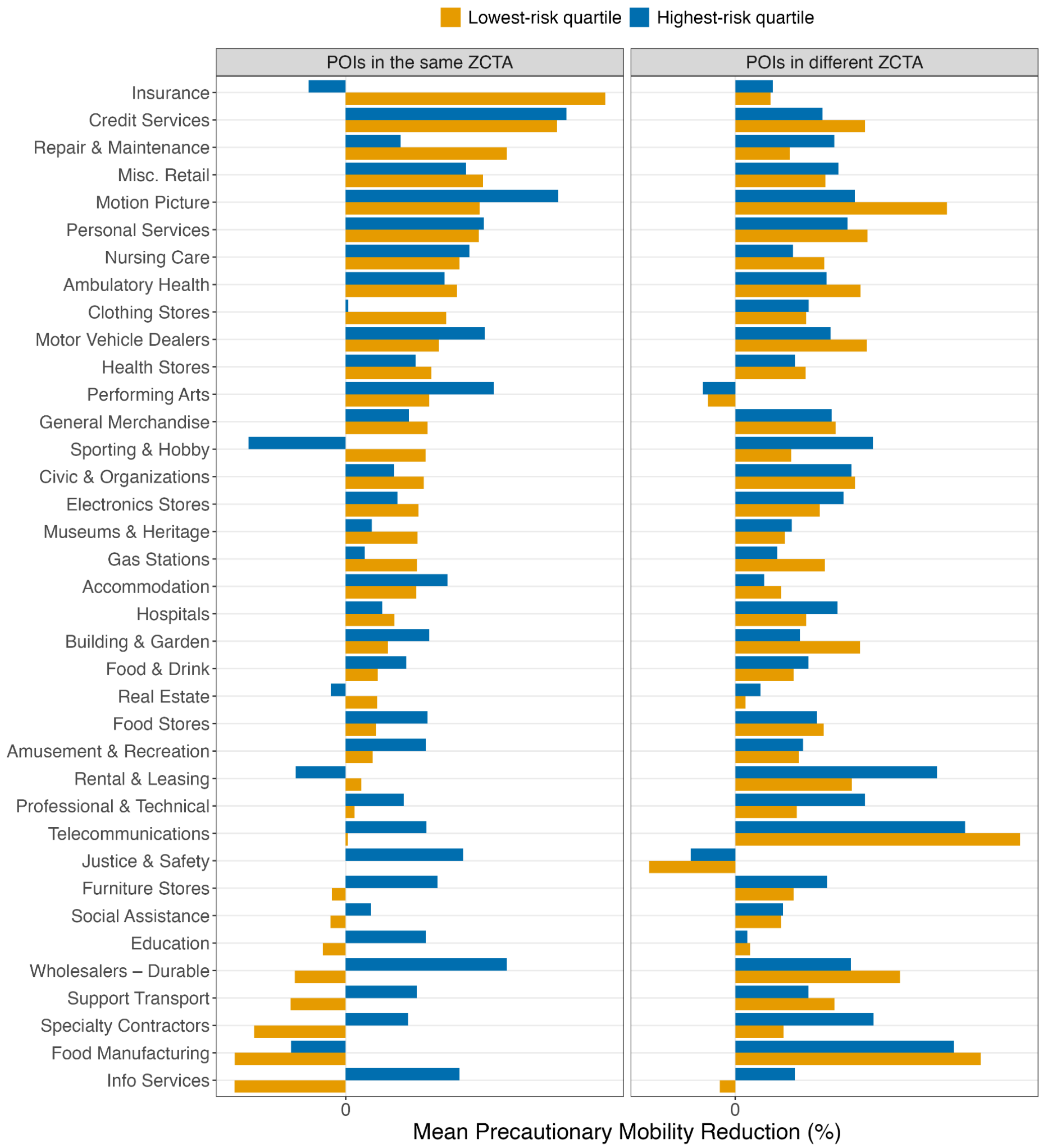

Figure S7. Mean precautionary mobility reduction (PMR) by place-of-interest (POI) category and community risk score quartile before the onset of statewide stay-at-home orders in Texas (February 3–March 15, 2020). Bars represent the mean PMR (percentage change relative to baseline) across ZIP Code Tabulation Areas (ZCTAs) in the lowest-risk and highest-risk community risk quartiles. The left panel shows visits to POIs located within the same ZCTA of residence, and the right panel shows visits to POIs located in different ZCTAs. Positive values indicate reductions in mobility relative to baseline, whereas negative values indicate increases.

Supplemental Material, Section 5: Characterization of ZIP codes excluded from analysis

ZCTAs were excluded from the mobility and hospitalization analyses at three distinct stages: during SVI computation, during the mobility completeness screen, and during linkage to hospitalization data. Table S3 summarizes the outcome for all 716 ZCTAs geographically located within the 20 most populous Texas counties.

Table S5. ZCTA exclusion reasons within the 20 most populous counties (n=716).

Exclusion stage	n	% of 716	Mean SVI	Median SVI	Mean population	Median population
Included in both mobility and hospitalization analyses	618	86.3%	0.528	0.520	32,691	29,034
SVI could not be computed (unstable/suppressed ACS estimates)	30	4.2%	—	—	1,020	213
SVI available, but no matching Safegraph mobility record	41	5.7%	0.481	0.474	2,165	1,010
Incomplete daily mobility tracking (≤320 of 333 days)	26	3.4%	0.580	0.638	7,990	6,649
Included in mobility analysis; no hospitalization data reported	2	0.3%	0.179	0.179	2,872	2,872
No visits recorded during the January 2020 baseline window	1	0.1%	0.814	0.814	2,027	2,027

Comparing ZCTAs included in the mobility analysis (n=618) against those excluded (n=98):

- **Community risk score (SVI):** no significant difference (Wilcoxon rank-sum test, W=20,852, p=0.92).
- **Population:** excluded ZCTAs were substantially smaller (median 1,253 vs. 28,909; Wilcoxon rank-sum test, $p<2.2\times10^{-16}$).
- **COVID-19 hospitalization rate (per 10,000 residents, February–July 2020):** no significant difference (median 19.8 vs. 19.9 per 10,000; Wilcoxon rank-sum test, p=0.38). Raw hospitalization counts differed significantly between groups ($p<2.2\times10^{-16}$), consistent with the population difference above rather than a difference in underlying risk.

ZIP-code exclusion at every stage of the analysis is attributable to population size and the resulting limits on mobile-device tracking coverage and ACS estimate stability, not to differences in community risk or COVID-19 hospitalization risk.
